# From post-war reconstruction to the twenty-first century – ophthalmic pathology in Freiburg 1945–2015. Part 2: review of 43,169 histological diagnoses from 39,256 specimens collected over 71 years at a large German tertiary eye care centre

**DOI:** 10.1186/s12886-025-04522-w

**Published:** 2026-01-16

**Authors:** Simone Nuessle, Mateusz Glegola, Tabea Schulz, Thomas Reinhard, Johannes Haedrich, Claudia Auw-Haedrich

**Affiliations:** 1 Eye Center at Medical Center, University of Freiburg, Killianstraße 5, 79106 Freiburg im Breisgau, Germany; 2 Eye Hospital, University Hospital Basel, Mittlere Str. 91, Basel, 4056 Switzerland; 3 Faculty of Medicine, University of Freiburg, Freiburg, Germany

**Keywords:** Ophthalmic pathology, Time trends, Chalazion, Basal cell carcinoma, Eyelid papilloma, Pterygium, Fuchs’ dystrophy

## Abstract

**Background:**

Ophthalmic pathology is essential for diagnosing ocular diseases, correlating clinical and histopathological findings, and advancing research. The Eye Center at the University of Freiburg, Germany, has archived histopathological specimens since 1945, offering a unique resource to analyse long-term diagnostic trends. This study examines 43,169 diagnoses from 39,256 specimens over 71 years (1945–2015), providing insights into the evolution of ophthalmic pathology at a major tertiary care centre.

**Methods:**

We performed a retrospective analysis of all archived ophthalmic pathology reports, categorising specimens by anatomical region and recording diagnoses, patient age and surgery dates. Data were analysed mostly in 10-year intervals, with annual sub-analyses for the four most frequent sites. Statistical parameters assessed changes in diagnostic frequency, patient demographics, and age-related trends. Results were compared with 38 international studies to contextualise findings.

**Results:**

The eyelid was the most common site (50%), with chalazion (18%), basal cell carcinoma (BCC) (16%), and papilloma (16%) as the leading diagnoses. The cornea (17%) was dominated by Fuchs’ dystrophy (19%), keratoconus (13%), and keratitis (11%), while pterygium (29%) and nevus (12%) prevailed in the conjunctiva (14%). In the orbit (1.2%), inflammation (12%) and lymphoma (9.5%) were most frequent. Key trends included a rise in chalazion, Fuchs’ dystrophy, and pterygium, linked to surgical advancements (e.g. microsurgery, DMEK) and increased UV exposure. The age range of patients widened for most diagnoses, reflecting an aging population and broader surgical indications. A decline in squamous cell carcinoma (SCC) and younger age at BCC diagnosis suggest improved UV protection and earlier detection. Regional comparisons revealed higher rates of chalazion and BCC in Freiburg than in Asian cohorts, likely due to genetic and environmental factors.

**Conclusions:**

This 71-year analysis highlights dynamic shifts in ophthalmic pathology, shaped by historical events, clinical progress, demographics, and environmental influences. The study emphasises the vital role of ophthalmologists in pathology, ensuring integrated clinical-histopathological expertise for accurate diagnoses and optimal patient outcomes. Our study data offer valuable insights into the frequency and evolving trends of the most common diagnoses over an extended period. These findings support future research in molecular diagnostics and global comparative studies, reinforcing the importance of ophthalmologist-led ophthalmic pathology in specialised eye care.

## Background

The Eye Center at the Medical Center, University of Freiburg, Germany, has a long-standing history in ophthalmic pathology that dates back to approximately 1870. Ophthalmic pathologists play a crucial role in providing accurate diagnoses for specimens excised from the eye and ocular adnexa. This precision is essential for establishing clinicopathological correlations and advancing ophthalmological research [1–4]. The field of ophthalmic pathology emerged as a sub-discipline of ophthalmology in the mid-nineteenth century [1]. Initially, pathology was based on gross tissue inspection. However, significant advancements in ophthalmic pathology were made with the invention of the ophthalmoscope by Hermann von Helmholtz in 1851 and the development of cellular pathology by Virchow in 1858. These innovations enabled the correlation of ocular cellular pathology with clinical images obtained through funduscopy [5]. Several distinguished ophthalmologists have significantly contributed to the advancement of ophthalmic pathology in Freiburg [6, 7].

In 1818, Carl Joseph Beck initiated the first series of lectures on ophthalmology in Freiburg, later founding the city’s inaugural surgical-ophthalmologic clinic in 1829. The field of ophthalmology in Germany was significantly advanced by Albrecht von Graefe, who established it as a distinct specialty separate from general surgery in 1857.

By 1868, the University of Freiburg had instituted a dedicated Chair of Ophthalmology. Wilhelm Manz, a collaborator of von Graefe in Berlin, was subsequently appointed as the first Professor of Ophthalmology in 1871. Around 1870, ophthalmic pathology emerged as a fundamental component of ophthalmology in Freiburg, culminating in the establishment of one of the earliest Eye Clinics in German-speaking regions in 1877. Theodor Axenfeld, renowned for his nearly 200 publications spanning all facets of ophthalmology and for the numerous eponymous terms attributed to him, took over the Chair of Ophthalmology in Freiburg in 1901.

The Freiburg Eye Clinic, however, was not exempt from the devastation of the Second World War. In November 1944, a sudden air raid by the Royal Air Force obliterated the entire University Hospital, including the Eye Clinic and all its histological sections and specimens collected before 1945. During these trying times, Franz Jankovsky, the caretaker of the Eye Clinic, played a crucial role in preserving the lab by salvaging and repairing essential equipment, thereby establishing the foundation for the new archive examined in the present study. Throughout this period, Wilhelm Wegner (1898–1967) served the longest tenure as director, from 1934 to 1967.

Special recognition must be given to Hanns-Hellmuth Unger (1919–2008), who began his career as an ophthalmology resident at the Freiburg Eye Clinic in 1949. His dedication to ophthalmopathological research culminated in his habilitation in 1957. In 1964, the newly constructed Freiburg Eye Centre, featuring a state-of-the-art ophthalmic pathology laboratory, was officially inaugurated. Three years later, in 1968, Günter Mackensen (1918–2000) assumed management, a position he held until 1987. Although not an ophthalmic pathologist by training, his profound interest in clinical science and the introduction of microsurgical techniques greatly advanced ophthalmic pathology in Freiburg. These innovations led to an increase in the frequency of biopsies taken from various anatomical regions [6].

Heinrich Witschel (1937–2019) succeeded as the director of the Freiburg Eye Clinic, serving from 1988 to 2002. He initially joined the ophthalmic pathology laboratory in Freiburg in 1971. Witschel further refined his expertise in ophthalmic pathology during a two-year period at the Armed Forces Institute of Pathology (AFIP) in Washington, USA (1974–75), under the mentorship of Lorenz Zimmerman (1920–2013). In 1992, Karin Loeffler joined Witschel as the head of the ophthalmic pathology laboratory. She later became a distinguished ophthalmic pathologist and served as the long-standing chairwoman of the *Association of German-speaking Ophthalmic Pathologists* (DOP).

Claudia Auw-Haedrich took over in 1996, succeeded by Simone Nuessle in 2024. Claudia Auw-Haedrich, who is the chairwoman of the DOP since 2023, initially honed her pathological expertise at the Institute of Clinical Pathology in Freiburg. This prepared her for her role as head of the Specialized Ophthalmic Pathology Laboratory at the Freiburg Eye Center. Thomas Reinhard, born in 1962 and specializing in corneal treatment and surgery, has served as director since 2003. That same year saw the establishment of the Lions Eye Bank in Freiburg. To this day, the Freiburg Eye Center maintains its own active ophthalmic pathology laboratory. In 2024, the lab received 4,294 specimens from 2,400 cases, with histopathological examinations conducted by ophthalmic pathologists who are also skilled eye surgeons.

Given the rich history of ophthalmic pathology in Freiburg, we decided to review its extensive archive, which spans over seven decades. While numerous studies have analyzed the full spectrum of histological diagnoses in specific ophthalmic locations, assessed diagnoses over particular periods, or examined time-dependent changes in selected anatomical areas [8–19], we are unaware of any study that has investigated long-term trends across the entire spectrum. Beginning in 2016, two doctoral theses were initiated to review and histologically re-evaluate all specimens in the archive, along with their associated findings, and enter them into a comprehensive database. We analyzed this large dataset, providing an extensive overview of 43,169 diagnoses from nearly 40,000 surgically obtained specimens, and mapping changes in the range of the most common ocular and periocular anatomical origins and their respective categories [20]. In this study, we analysed all diagnoses from our previous work, omitting only those previously assessed, to ensure comprehensive coverage of the diagnostic spectrum. This approach facilitates a deeper exploration of long-term trends and age-related changes in the frequency of the most common diagnoses.

## Methods

At our ophthalmic pathology laboratory, we specialize in the preparation and examination of histological samples. These primarily include surgical resection specimens and both excisional and incisional biopsies, mainly sourced from our in-house eye clinic. Since the mid-1990s, we have expanded our reach to accept samples from pathology laboratories, eye hospitals, and practicing ophthalmologists across Germany and beyond. Each sample undergoes meticulous processing for light microscopy, adhering to standard protocols, with select specimens receiving special staining treatments including immunohistochemistry (IHC). Furthermore, certain samples are analyzed using transmission electron microscopy to ensure comprehensive evaluation. Our team of seasoned ophthalmic pathologists, who also possess extensive experience as eye surgeons, utilize the latest histopathological criteria to deliver precise diagnoses. Our experts adhere to rigorous standards through ongoing professional development, including active participation in both national and international ophthalmic pathology meetings. Of note, three consultants are—or have been—active members of the *European Ophthalmic Pathology Society* (EOPS). We facilitate consultations at both national and international levels as required.

For this study, we identified the ophthalmic pathology reports of all histological samples archived at the Eye Center, Medical Center, University of Freiburg, spanning from 1945 to 2015. We conducted a retrospective analysis of all histopathological diagnoses detailed in these archived reports, categorizing them based on their topographical regions. A significant number of slides across our study period underwent rigorous, time-independent review and were recognized for their exceptional quality. These slides were also presented at prestigious international conferences, including EOPS and joint meetings with the US-based *Verhoeff-Zimmermann Society* (VZS). Original diagnoses were confirmed in over 99% of cases. Any necessary revisions were subject to a comprehensive assessment of their potential impact on patient treatment and outcomes. Critically, none of these revisions influenced the standard of care or clinical management for the affected patients. It is important to note that some specimens had multiple diagnoses, leading to a higher count of diagnoses than the number of collected samples. Not discussed in this study are temporal arteries, as their detailed analysis has already been published in our overview of the topographical regions [20]. We opted not to further analyse lens specimens, given that nearly all cases involved cataracts. Similarly, we did not include eyeball and evisceration samples due to the predominantly descriptive nature of their ophthalmic reports, which do not represent a primary histological diagnosis.

To explore the progression of underlying diseases during the study period (1945 to 2015) that necessitated surgical removal and histopathological examination, we established intervals of ten years, with the exception of the final period, which spanned eleven years. Furthermore, we implemented annual intervals for the four most frequent anatomical locations, chosen for their higher sample numbers. This approach facilitated a more detailed analysis of the development of the five most common diagnoses over time and allowed for more adaptable and precise interval adjustments, enhancing comparison with findings from existing literature.

Using Excel® 2019 (Microsoft, Redmond, WA, USA), we recorded histopathological diagnoses, topographical regions, patient ages, and surgery dates into a comprehensive database. To assess the most prevalent diagnoses for each ophthalmic topographical region and their evolution over time—including variations in patient age at the time of surgery—we employed statistical parameters such as mean, median, and range. To enable direct comparison with the existing literature, we calculated the relative frequencies of the most common diagnoses at the predominant anatomical sites, aligning our time periods with those of 38 referenced studies. We systematically reviewed published data—both absolute counts and proportions—across all relevant localisations. Where data were not explicitly reported, we conducted detailed analyses to classify diagnoses according to their respective localisations and diagnostic categories.

## Results

Over a 71-year period, our study analysed 43,169 histopathological diagnoses from 39,256 archived specimens. The most frequent regions (1945–2015) were distributed as follows: eyelid (*n* = 21,764; 50%), cornea (*n* = 7,319; 17%), conjunctiva (*n* = 5,963; 14%), eyeball (*n* = 3,555; 8.2%), temporal artery (*n* = 1,517; 3.5%), and orbit (*n* = 535; 1.2%). Less frequent regions included the lens (*n* = 370; 0.86%), lacrimal duct (*n* = 303; 0.70%), uvea (*n* = 202; 0.51%), unspecified intraocular tissue (*n* = 198; 0.46%), vitreous (*n* = 178; 0.41%), non-ophthalmological locations (*n* = 173; 0.40%), such as the nose and forehead. Other regions comprised the lacrimal gland (*n* = 144; 0.33%), retina (*n* = 93; 0.22%), exenteration (*n* = 59; 0.14%), evisceration (*n* = 60; 0.14%), sclera (*n* = 38; 0.088%), anterior chamber angle (*n* = 37; 0.086%), anterior chamber (*n* = 27; 0.063%) and optic nerve (*n* = 15; 0.035%). Some samples could not be categorised and were classified as “unspecified” (*n* = 601; 1.4%). Note that percentages may not always sum to 100 due to rounding.

**Fig. 1:**
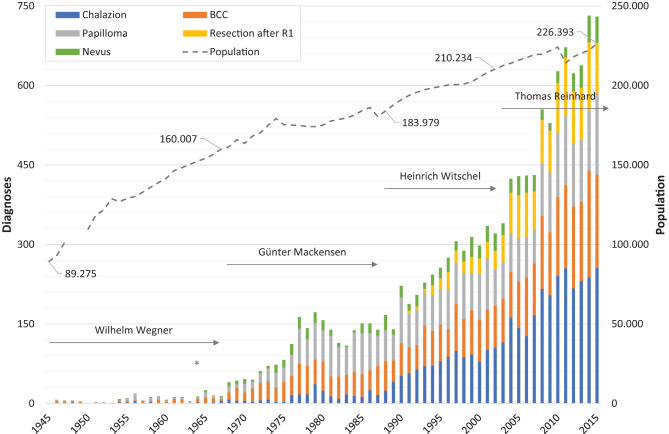
*Annual number of specimens for the most frequent eyelid diagnoses.* This figure illustrates the changes in the annual numbers of the five most frequent eyelid diagnoses over the study period (1945–2015). The duration of medical directorships is indicated as follows: Wegner (1934 to 1967), Mackensen (1968 to 1987), Witschel (1988 to 2002), and Reinhard (since 2003). The commissioning of the new Freiburg Eye Center in 1964 is marked with an asterisk (*). Additionally, the dashed line represents the demographic development in the city of Freiburg, showing the number of inhabitants (note: data unavailable for 1948–1949). BCC: basal cell carcinoma

**Fig. 2:**
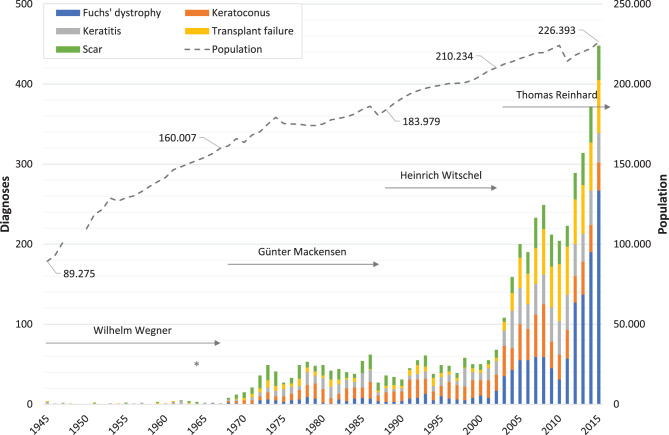
*Annual number of specimens for the most frequent corneal diagnoses.* This figure illustrates the changes in the annual numbers of the five most frequent corneal diagnoses over the study period (1945–2015). The duration of medical directorships is indicated as follows: Wegner (1934 to 1967), Mackensen (1968 to 1987), Witschel (1988 to 2002), and Reinhard (since 2003). The commissioning of the new Freiburg Eye Center in 1964 is marked with an asterisk (*). Additionally, the dashed line represents the demographic development in the city of Freiburg, showing the number of inhabitants (note: data unavailable for 1948–1949)

**Fig. 3:**
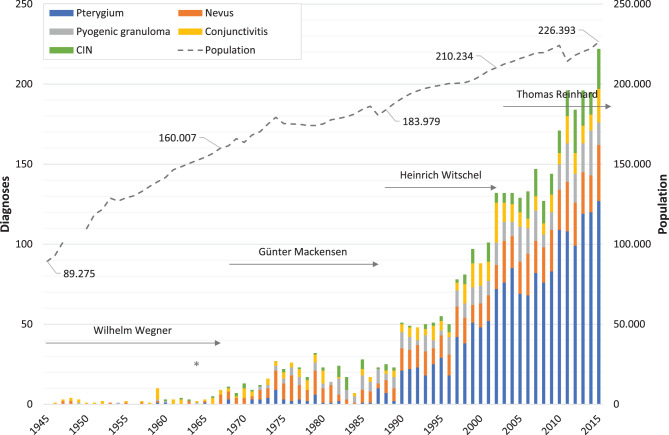
*Annual number of specimens for the most frequent conjunctival diagnoses.* This figure illustrates the changes in the annual numbers of the five most frequent conjunctival diagnoses over the study period (1945–2015). The duration of medical directorships is indicated as follows: Wegner (1934 to 1967), Mackensen (1968 to 1987), Witschel (1988 to 2002), and Reinhard (since 2003). The commissioning of the new Freiburg Eye Center in 1964 is marked with an asterisk (*). Additionally, the dashed line represents the demographic development in the city of Freiburg, showing the number of inhabitants (note: data unavailable for 1948–1949). CIN: conjunctival intraepithelial neoplasia

**Fig. 4:**
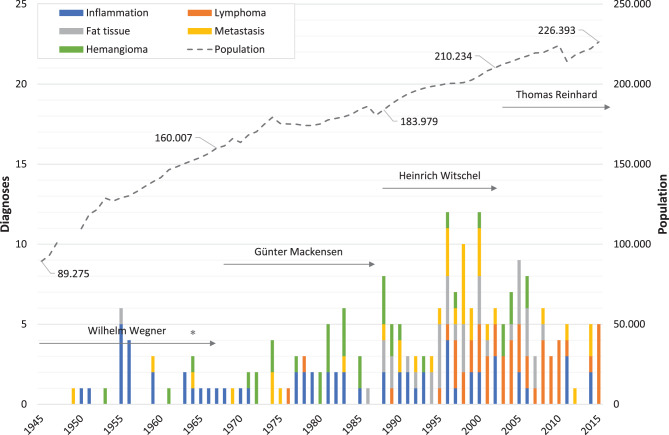
*Annual number of specimens for the most frequent orbital diagnoses. *This figure illustrates the changes in the annual numbers of the five most frequent orbital diagnoses over the study period (1945–2015). The duration of medical directorships is indicated as follows: Wegner (1934 to 1967), Mackensen (1968 to 1987), Witschel (1988 to 2002), and Reinhard (since 2003). The commissioning of the new Freiburg Eye Center in 1964 is marked with an asterisk (*). Additionally, the dashed line represents the demographic development in the city of Freiburg, showing the number of inhabitants (note: data unavailable for 1948–1949)

Figures 1, 2, 3, 4 illustrate the annual variations in the five most frequent diagnoses for eyelid, corneal, conjunctival, and orbital conditions, respectively, over the study period from 1945 to 2015. Table 1 presents an overview of the most common diagnoses, listing the “Top 10” for the four primary topographical areas and the “Top 5” for the less frequent regions within each interval, arranged in descending order. Some intervals may include more than 10 or 5 diagnoses due to ties in ranking. The table displays both the numbers and their relative frequencies, highlighting changes in the ranking lists over time for each interval and location throughout the observation period.

**Fig. 5:**
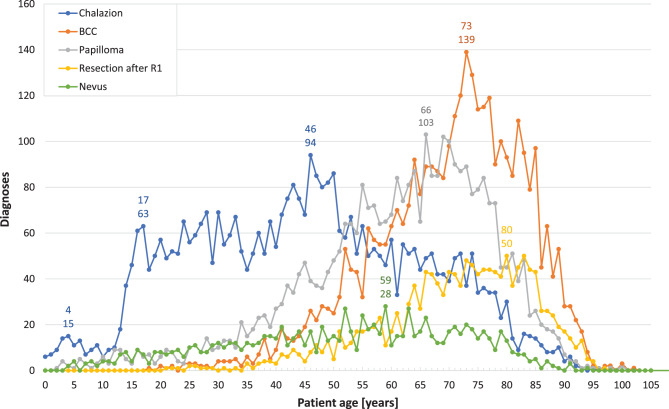
*Distributions of patient ages for the most frequent eyelid diagnoses.* This figure presents the frequency distribution of patient ages at the time of surgery for the five most common eyelid diagnoses over the study period (1945–2015). BCC: basal cell carcinoma

**Fig. 6:**
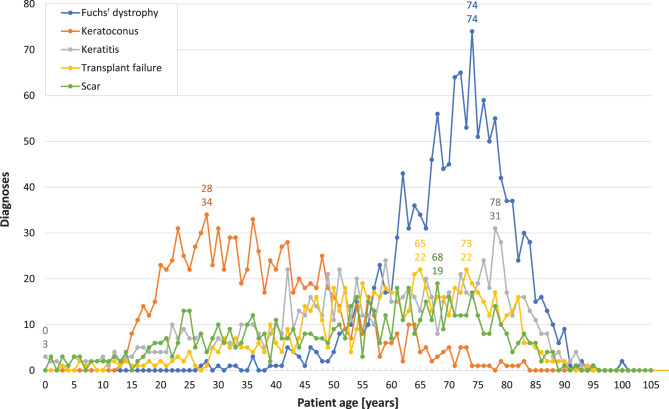
*Distributions of patient ages for the most frequent corneal diagnoses.* This figure presents the frequency distribution of patient ages at the time of surgery for the five most common corneal diagnoses over the study period (1945–2015)

**Fig. 7:**
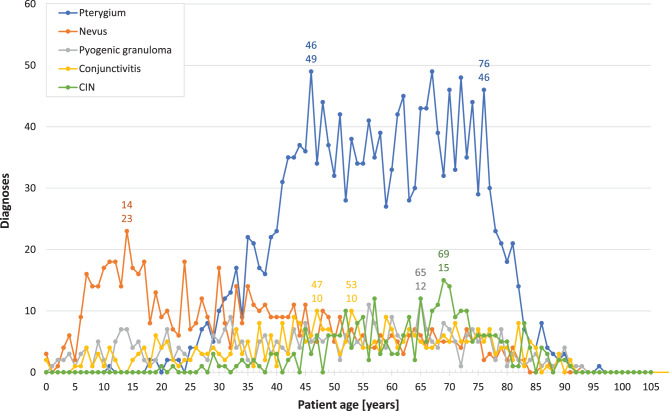
*Distributions of patient ages for the most frequent conjunctival diagnoses.* This figure presents the frequency distribution of patient ages at the time of surgery for the five most common conjunctival diagnoses over the study period (1945–2015). CIN: conjunctival intraepithelial neoplasia

**Fig. 8:**
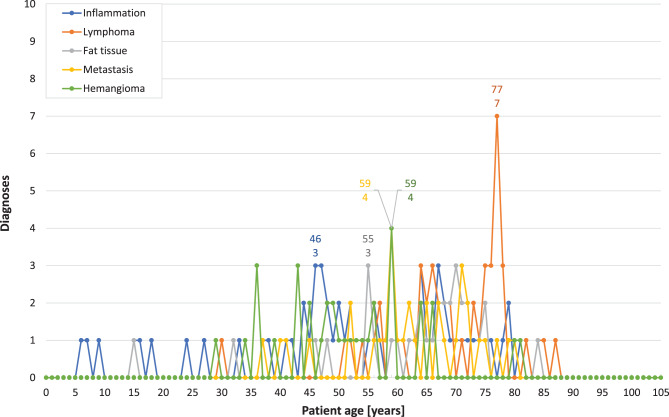
*Distributions of patient ages for the most frequent orbital diagnoses.* This figure presents the frequency distribution of patient ages at the time of surgery for the five most common orbital diagnoses over the study period (1945–2015)

The frequency distributions of the five most common diagnoses by recorded patient age at surgery over the entire period are illustrated in Fig. 5 for eyelid conditions, Fig. 6 for corneal conditions, Fig. 7 for conjunctival conditions, and Fig. 8 for orbital conditions. Table 2 enumerates diagnoses that consistently ranked within the “Top 10” or “Top 5” throughout the observation period. Additionally, the shifts in patients’ ages at the time of surgery are indicated for each specified time interval.

**Table 1:** Frequency and trends in common diagnoses by topographical region

	1945–1954		1955–1964		1965–1974		1975–1984		1985–1994		1995–2004		2005–2015	
**Eyelid** (*n* = 21,764)	SCC	18 (22%)	Papilloma	46 (22%)	BCC	196 (26%)	Papilloma	570 (26%)	Papilloma	655 (22%)	Chalazion	1,008 (20%)	Chalazion	2,293 (22%)
	BCC	17 (21%)	BCC	43 (20%)	Papilloma	135 (18%)	BCC	450 (20%)	BCC	531 (18%)	Papilloma	772 (16%)	BCC	1,541 (15%)
	Papilloma	12 (14%)	SCC	25 (12%)	Nevus	58 (7.8%)	Chalazion	166 (7.5%)	Chalazion	432 (14%)	BCC	769 (16%)	Papilloma	1,188 (11%)
	Chalazion	7 (8.4%)	Chalazion	12 (5.5%)	Epidermal cyst	47 (6.3%)	Nevus	142 (6.4%)	Nevus	177 (5.9%)	Resection after R1	351 (7.1%)	Resection after R1	1,025 (10%)
	Molluscum contagiosum	5 (6.0%)	Granuloma	10 (4.8%)	Chalazion	44 (5.9%)	Epidermal cyst	135 (6.1%)	Hidrocystoma	123 (4.1%)	Nevus	257 (5.2%)	Hidrocystoma	433 (4.1%)
	Xanthelasma	5 (6.0%)	Nevus	9 (4.3%)	Dermoid cyst	36 (4.8%)	Dermoid cyst	64 (2.9%)	Epidermal cyst	115 (3.8%)	Hidrocystoma	144 (2.9%)	Nevus	349 (3.3%)
	Epidermal cyst	4 (4.8%)	Epidermal cyst	8 (3.8%)	Hemangioma	20 (2.7%)	Hidrocystoma	47 (2.1%)	Dermoid cyst	60 (2.0%)	Epidermal cyst	120 (2.4%)	Xanthelasma	305 (2.9%)
	Dermoid cyst	2 (2.4%)	Actinic keratosis	6 (2.9%)	Granuloma	13 (1.7%)	Hemangioma	34 (1.5%)	Granuloma	56 (1.9%)	Dermoid cyst	80 (1.6%)	Epidermal cyst	299 (2.8%)
	Fibroma	2 (2.4%)	Dermoid cyst	5 (2.4%)	Neurofibroma	9 (1.2%)	Granuloma	33 (1.5%)	Hemangioma	52 (1.7%)	Xanthelasma	78 (1.6%)	Blepharitis	191 (1.8%)
	Granuloma	2 (2.4%)	Granulation tissue	4 (1.9%)	Molluscum contagiosum	8 (1.1%)	Xanthelasma	31 (1.4%)	Resection after R1	39 (1.3%)	Blepharitis	76 (1.5%)	Malposition	153 (1.4%)
	Nevus	2 (2.4%)	Hemangioma	4 (1.9%)	SCC	8 (1.1%)								
					Xanthelasma	8 (1.1%)								
	Other diagnoses	7 (8.4%)	Other diagnoses	38 (18%)	Other diagnoses	164 (22%)	Other diagnoses	534 (24%)	Other diagnoses	769 (26%)	Other diagnoses	1,287 (26%)	Other diagnoses	2,791 (26%)
	Sum	83	Sum	210	Sum	746	Sum	2,206	Sum	3,009	Sum	4,942	Sum	10,568
**Cornea** (*n* = 7,319)	Ulcer	7 (26%)	Inflammation	13 (27%)	Scar	76 (29%)	Keratoconus	122 (22%)	Keratoconus	155 (19%)	Keratoconus	217 (18%)	Fuchs’ dystrophy	1,082 (25%)
	Inflammation	6 (22%)	Scar	7 (14%)	Keratoconus	33 (13%)	Inflammation	107 (19%)	BK w	114 (14%)	Inflammation	165 (14%)	Transplant failure	603 (14%)
	Transplant failure	4 (15%)	Dermoid	6 (12%)	Inflammation	29 (11%)	Scar	89 (16%)	Scar	101 (12%)	Fuchs’ dystrophy	150 (12%)	Keratoconus	446 (10%)
	SCC	4 (15%)	Infection	4 (8.2%)	Transplant failure	28 (11%)	Transplant failure	55 (10%)	Inflammation	77 (9.4%)	BK w/o	128 (11%)	Inflammation	435 (10%)
	Degeneration	2 (7.4%)	Ulcer	4 (8.2%)	Fuchs’ dystrophy	21 (8.1%)	Fuchs’ dystrophy	50 (8.8%)	Fuchs’ dystrophy	62 (7.6%)	Ulcer	93 (7.6%)	Scar	368 (8.4%)
	Scar	2 (7.4%)	Degeneration	3 (6.1%)	Infection	10 (3.8%)	BK w	29 (5.1%)	Transplant failure	48 (5.9%)	Scar	87 (7.2%)	BK w/o	288 (6.6%)
	Epithelial defect	1 (3.7%)	Transplant failure	3 (6.1%)	Ulcer	7 (2.7%)	Ulcer	19 (3.3%)	Ulcer	35 (4.3%)	Transplant failure	65 (5.3%)	Ulcer	246 (5.6%)
	Melanoma	1 (3.7%)	Keratoconus	2 (4.1%)	Dermoid	6 (2.3%)	BK w/o	9 (1.6%)	Infection	26 (3.2%)	Infection	52 (4.3%)	Salzmann	189 (4.3%)
			CIN	1 (2.0%)	BK w	4 (1.5%)	Macular dystrophy	9 (1.6%)	BK w/o	23 (2.8%)	BK w	39 (3.0%)	Infection	154 (3.5%)
			Cyst	1 (2.0%)	Granular dystrophy	4 (1.5%)	Infection	7 (1.2%)	Macular dystrophy	12 (1.5%)	Salzmann	25 (2.1%)	BK w	79 (1.8%)
			Epithelial hyperplasia	1 (2.0%)										
			Limbal papillae	1 (2.0%)										
			Melanoma	1 (2.0%)										
			Normal	1 (2.0%)										
			SCC	1 (2.0%)										
	Other diagnoses	0	Other diagnoses	0	Other diagnoses	42 (16%)	Other diagnoses	72 (13%)	Other diagnoses	164 (20%)	Other diagnoses	195 (16%)	Other diagnoses	492 (11%)
	Sum	27	Sum	49	Sum	260	Sum	568	Sum	817	Sum	1,216	Sum	4,382
**Conjunctiva** (*n* = 5,963)	Conjunctivitis	10 (21%)	Conjunctivitis	17 (20%)	Nevus	46 (16%)	Nevus	85 (20%)	Pterygium	130 (21%)	Pterygium	511 (31%)	Pterygium	1,060 (37%)
	Granulation	5 (10%)	Granuloma	13 (16%)	Cyst	30 (10%)	Papilloma	43 (10%)	Nevus	99 (16%)	Nevus	165 (10%)	Nevus	281 (10%)
	Granuloma	5 (10%)	Papilloma	10 (12%)	Conjunctivitis	27 (9.4%)	Pyogenic granuloma	43 (10%)	Pyogenic granuloma	71 (11%)	Conjunctivitis	115 (7.0%)	Pyogenic granuloma	194 (6.8%)
	Nevus	5 (10%)	Melanoma	5 (6.0%)	Papilloma	27 (9.4%)	Conjunctivitis	34 (7.9%)	Conjunctivitis	45 (7.2%)	Pyogenic granuloma	99 (6.0%)	CIN	188 (6.6%)
	SCC	5 (10%)	Nevus	5 (6.0%)	Pterygium	23 (8.0%)	Cyst	27 (6.3%)	Papilloma	39 (6.2%)	Cyst	62 (3.8%)	Conjunctivitis	121 (4.2%)
	Cyst	3 (6.3%)	Cyst	4 (4.8%)	Granuloma	15 (5.2%)	CIN	23 (5.3%)	Cyst	32 (5.1%)	CIN	56 (3.4%)	Pinguecula	107 (3.8%)
	Papilloma	3 (6.3%)	SCC	4 (4.8%)	Pinguecula	12 (4.2%)	Pterygium	20 (4.6%)	Scar tissue	26 (4.1%)	Scar tissue	55 (3.4%)	Cyst	96 (3.4%)
	Pinguecula	3 (6.3%)	CIN	3 (3.6%)	CIN	9 (3.1%)	Melanoma	18 (4.2%)	CIN	20 (3.2%)	Pinguecula	54 (3.3%)	Melanosis	76 (2.7%)
	Pyogenic granuloma	2 (4.2%)	Pyogenic granuloma	3 (3.6%)	Granulation	8 (2.8%)	Granuloma	11 (2.6%)	Pinguecula	19 (3.0%)	Melanosis	45 (2.7%)	Papilloma	66 (2.3%)
	Fibroma	1 (2.1%)	Lymphoma	2 (2.4%)	Pyogenic granuloma	7 (2.4%)	SCC	11 (2.6%)	Melanosis	15 (2.4%)	Papilloma	44 (2.7%)	Normal	65 (2.3%)
	Hemangioma	1 (2.1%)	Metastasis	2 (2.4%)										
	Hyperplasia	1 (2.1%)	Pingucula	2 (2.4%)										
	Melanoma	1 (2.1%)	Pterygium	2 (2.4%)										
	Oncocytoma	1 (2.1%)												
	Sebaceous adenoma	1 (2.1%)												
	Sebaceous hyperplasia	1 (2.1%)												
	Other diagnoses	0	Other diagnoses	11 (13%)	Other diagnoses	83 (29%)	Other diagnoses	116 (27%)	Other diagnoses	132 (21%)	Other diagnoses	431 (26%)	Other diagnoses	595 (21%)
	Sum	48	Sum	83	Sum	287	Sum	431	Sum	628	Sum	1,637	Sum	2.,849
**Orbit** (*n* = 535)	Connective tissue	2 (14%)	Inflammation	14 (42%)	Fibrotic muscle	7 (11%)	Inflammation	12 (13%)	Granuloma	11 (11%)	Lymphoma	24 (17%)	Lymphoma	24 (28%)
	Inflammation	2 (14%)	Normal muscle	4 (12%)	Normal muscle	7 (11%)	Hemangioma	9 (10%)	Inflammation	10 (10%)	Metastasis	17 (12%)	Fat tissue	11 (13%)
	Dermoid cyst	1 (7.1%)	Granuloma	2 (6.1%)	Inflammation	6 (9.2%)	Melanoma	6 (6.6%)	Fat tissue	9 (8.7%)	Fat tissue	15 (10%)	Inflammation	9 (11%)
	Granuloma	1 (7.1%)	Hemangioma	2 (6.1%)	Muscular dystrophy	6 (9.2%)	Connective tissue	4 (4.4%)	Hemangioma	9 (8.7%)	Inflammation	12 (8.3%)	Scar	8 (9.4%)
	Hemangioma	1 (7.1%)	Meningeoma	2 (6.1%)	Hemangioma	5 (7.7%)	Meningeoma	4 (4.4%)	Varix	7 (6.8%)	Dermoid cyst	7 (4.9%)	Granuloma	7 (8.2%)
	Hematoma	1 (7.1%)	Metastasis	2 (6.1%)	Metastasis	3 (4.6%)	Dermoid cyst	3 (3.3%)	Metastasis	5 (4.9%)	Hemangioma	7 (4.9%)	Metastasis	5 (5.9%)
	Hyperplastic lymph node	1 (7.1%)	Sarcoma	2 (6.1%)	Dermoid cyst	2 (3.1%)	FH	3 (3.3%)	Lymphangioma	4 (3.9%)	Normal muscle	7 (4.9%)	Melanoma	3 (3.5%)
	Leucemia	1 (7.1%)	Scar	2 (6.1%)	Granuloma	2 (3.1%)	Granulation	3 (3.3%)	Normal muscle	4 (3.9%)	Schwannoma	6 (4.2%)	Cyst	2 (2.4%)
	Lymphoma	1 (7.1%)	Dermoid cyst	1 (3.0%)	Melanoma	2 (3.1%)	MFH	3 (3.3%)	SCC	4 (3.9%)	Granuloma	3 (2.1%)	Hemangioma	2 (2.4%)
	Metastasis	1 (7.1%)	Fat tissue	1 (3.0%)	Meningeoma	2 (3.1%)	Rhabdo-myosarcoma	3 (3.3%)	Connective tissue	3 (2.9%)	Lymphangioma	3 (2.1%)	Langerhans cell histiocytosis	2 (2.4%)
	Necrosis	1 (7.1%)	Osteoblastoma	1 (3.0%)	Mucocele	2 (3.1%)			Dermoid cyst	3 (2.9%)			Schwannoma	2 (2.4%)
	Unknown tumour	1 (7.1%)							Schwannoma	3 (2.9%)				
	Other diagnoses	0	Other diagnoses	0	Other diagnoses	21 (32%)	Other diagnoses	41 (45%)	Other diagnoses	31 (30%)	Other diagnoses	43 (30%)	Other diagnoses	10 (12%)
	Sum	14	Sum	33	Sum	65	Sum	91	Sum	103	Sum	144	Sum	85
**Lacrimal duct system **(*n* = 303)	Dacryocystitis	23 (72%)	Dacryocystitis	42 (89%)	Dacryocystitis	11 (50%)	Dacryocystitis	16 (43%)	Dacryocystitis	10 (33%)	Dacryocystitis	32 (46%)	Dacryocystitis	27 (40%)
	Tuberculosis	3 (9.4%)	Carcinoma	2 (4.3%)	Canaliculitis	3 (14%)	Canaliculitis	9 (26%)	Canaliculitis	5 (17%)	Lacrimal sac concrement	17 (24%)	Canaliculitis	20 (30%)
	SCC	2 (6.3%)	Connective tissue	1 (2.1%)	Lacrimal sac concrement	2 (9.1%)	Lacrimal sac soncrement	6 (11%)	Scar	5 (17%)	Canaliculitis	6 (8.6%)	Lacrimal sac concrement	10 (15%)
	Granulation	1 (3.1%)	Cyst	1 (2.1%)	Melanoma	2 (9.1%)	Scar	2 (5.7%)	Lacrimal sac concrement	4 (13%)	Normal	5 (7.1%)	Lymphoma	3 (4.5%)
	Normal	1 (3.1%)	Lacrimal sac concrement	1 (2.1%)	Normal	2 (9.1%)	Fistula	1 (2.9%)	Normal	2 (6.7%)	Lymphoma	4 (5.7%)	Normal	3 (4.5%)
	Post-inflammation	1 (3.1%)			Scar	1 (4.5%)	Lymphatic hyperplasia	1 (2.9%)			Benign Tumour	1 (1.4%)	Fistula	2 (3.0%)
	Scar	1 (3.1%)			Presaccal stenosis	1 (4.5%)					Diverticula	1 (1.4%)		
											Fistula	1 (1.4%)		
											Granulation	1 (1.4%)		
											Mucoepidermoid-carcinoma	1 (1.4%)		
											Scar	1 (1.4%)		
	Other diagnoses	0	Other diagnoses	0	Other diagnoses	0	Other diagnoses	0	Other diagnoses	4 (13%)	Other diagnoses	0	Other diagnoses	2 (3.0%)
	Sum	32	Sum	47	Sum	22	Sum	35	Sum	30	Sum	70	Sum	67
**Uvea** (*n* = 220)	Iritis	7 (39%)	Post-iritis	3 (25%)	Iris melanoma	7 (26%)	Iris melanoma	7 (18%)	Normal Iris	5 (24%)	Normal Iris	18 (30%)	Iris melanoma	8 (19%)
	Iris atrophy	3 (17%)	Iris melanoma	2 (17%)	Iritis	6 (22%)	Iris nevus	3 (7.5%)	Ciliary body melanoma	2 (9.5%)	Iris melanoma	10 (17%)	Normal iris	7 (17%)
	Iris nevus	3 (17%)	Iritis	2 (17%)	Iris cyst	4 (15%)	Normal choroid	3 (7.5%)	Iris melanoma	2 (9.5%)	Ciliary body melanoma	7 (12%)	Iritis	7 (17%)
	Choroidal fibrosarcoma	1 (5.6%)	Iris atrophy	1 (8.3%)	Normal Iris	3 (11%)	Normal iris	3 (7.5%)	Iris nevus	2 (9.5%)	Iritis	5 (8.3%)	Choroidal melanoma	4 (10%)
	Iris edema	1 (5.6%)	Normal ciliary body	1 (8.3%)	Ciliary body adenoma	1 (3.7%)	Rubeosis iridis	3 (7.5%)	Necrotis Iris	2 (9.5%)	Iris nevus	4 (6.7%)	Ciliary body melanoma	2 (4.8%)
	Iris melanoma	1 (5.6%)	Normal iris	1 (8.3%)	Iris fibroma	1 (3.7%)							Iris nevus	2 (4.8%)
	Lisch nodule	1 (5.6%)	Thickened iris stroma	1 (8.3%)	Iris melanocytoma	1 (3.7%)							Iris prolapse	2 (4.8%)
	Normal Iris	1 (5.6%)	Xanthogranuloma	1 (8.3%)	Irishyperplasia	1 (3.7%)								
					Uveal foreign body granuloma	1 (3.7%)								
					Uveal hyperemia	1 (3.7%)								
					Vascularised iris	1 (3.7%)								
	Other diagnoses	0	Other diagnoses	0	Other diagnoses	0	Other diagnoses	21 (53%)	Other diagnoses	8 (38%)	Other diagnoses	16 (27%)	Other diagnoses	10 (24%)
	Sum	18	Sum	12	Sum	27	Sum	40	Sum	21	Sum	60	Sum	42
**Intraocular tissue, unspecified** (*n* = 198)	-		-		Trauma-associated without retina	2 (100%)	Trauma-associated without retina	1 (100%)	Trauma-associated without retina	29 (67%)	Trauma-associated without retina	60 (73%)	Trauma-associated without retina	54 (77%)
									Trauma-associated with retina	14 (33%)	Trauma-associated with retina	21 (26%)	Trauma-associated with retina	14 (20%)
											Bacterial endophthalmitis	1 (1.2%)	Fibrin	1 (1.4%)
													Purulent endophthalmitis	1 (1.4%)
	Other diagnoses	0	Other diagnoses	0	Other diagnoses	0	Other diagnoses	0	Other diagnoses	0	Other diagnoses	0	Other diagnoses	0
	Sum	0	Sum	0	Sum	2	Sum	1	Sum	43	Sum	82	Sum	70
**Vitreous** (*n* = 178)	-		Normal	1 (100%)	Normal	1 (50%)	Vitritis	2 (33%)	Vitritis	13 (52%)	Vitritis	36 (49%)	Vitritis	32 (45%)
					Pigmented connective tissue	1 (50%)	Blood	1 (17%)	Normal	4 (16%)	Normal	9 (12%)	Normal	11 (15%)
							Foreign body granuloma	1 (17%)	Blood	3 (12%)	PHPV	5 (6.8%)	Lymphoma not ruled out	7 (10%)
							Normal	1 (17%)	Oxalate crystals	2 (8.0%)	Blood	4 (5.5%)	Lymphoma	6 (8.5%)
							PHPV	1 (17%)	PHPV	2 (8.0%)	Lymphoma	4 (5.5%)	Blood	3 (4.2%)
	Other diagnoses	0	Other diagnoses	0	Other diagnoses	0	Other diagnoses	0	Other diagnoses	1 (4.0%)	Other diagnoses	15 (21%)	Other diagnoses	12 (17%)
	Sum	0	Sum	1	Sum	2	Sum	6	Sum	25	Sum	73	Sum	71
**Lacrimal gland** (*n* = 144)	Hypertrophy	2 (100%)	Lymphocytic	2 (40%)	Pleomorphic adenoma	5 (42%)	Dacryoadenitis	9 (38%)	Pleomorphic adenoma	6 (32%)	Dacryoadenitis	21 (57%)	Normal	13 (29%)
			Normal	2 (40%)	Carcinoma	2 (17%)	Pleomorphic adenoma	4 (17%)	Dacryoadenitis	4 (21%)	Pleomorphic adenoma	4 (11%)	Cyst	13 (29%)
			Cyst	1 (20%)	Cyst	1 (8.3%)	Carcinoma	3 (13%)	Normal	4 (21%)	Carcinoma	3 (8.1%)	Dacryoadenitis	5 (11%)
					Dacryoadenitis	1 (8.3%)	Cyst	3 (13%)	Carcinoma	3 (16%)	Normal	3 (8.1%)	Lymphoma	4 (8.9%)
					Lymphoma	1 (8.3%)	Connective tissue	2 (8.3%)	Cyst	1 (5.3%)	Lymphoma	2 (5.4%)	Lymphatic hyperplasia	2 (4.4%)
					Malignant tumour, undefined	1 (8.3%)			Hypertrophy	1 (5.3%)			Pleomorphic adenoma	2 (4.4%)
					PICPS	1 (8.3%)								
	Other diagnoses	0	Other diagnoses	0	Other diagnoses	0	Other diagnoses	3 (13%)	Other diagnoses	0	Other diagnoses	4 (11%)	Other diagnoses	6 (13%)
	Sum	2	Sum	5	Sum	12	Sum	24	Sum	19	Sum	37	Sum	45
**Retina** (*n* = 93)	-		Glioma	1 (100%)	Normal	1 (50%)	Connective tissue	1 (100%)	Proliferative vitreoretinopathy	3 (33%)	CNV	14 (41%)	Epiretinal gliosis	9 (20%)
					Degenerative	1 (50%)			Blood	2 (22%)	Epiretinal gliosis	6 (18%)	CNV	7 (15%)
									Epiretinal gliosis	1 (13%)	Proliferative vitreoretinopathy	4 (12%)	Proliferative diabetic membrane	6 (13%)
									Malignant astrocytoma	1 (13%)	Unspecific membrane	3 (8.8%)	Proliferative vitreoretinopathy	6 (13%)
									Normal	1 (13%)	Blood	2 (5.9%)	Granuloma	4 (8.7%)
									Retinoblastoma	1 (13%)			Scar	4 (8.7%)
	Other diagnoses	0	Other diagnoses	0	Other diagnoses	0	Other diagnoses	0	Other diagnoses	0	Other diagnoses	5 (15%)	Other diagnoses	10 (22%)
	Sum	0	Sum	1	Sum	2	Sum	1	Sum	9	Sum	34	Sum	46
**Exenteration** (*n* = 59)	Metastasis	3 (33%)	SCC	2 (50%)	Lymphoma	3 (43%)	Rhabdomyosarcoma	3 (38%)	BCC	7 (47%)	Melanoma	2 (25%)	Resection after R1	2 (25%)
	BCC	1 (11%)	Perforating trauma	1 (25%)	Acute inflammation	2 (29%)	Normal	2 (25%)	SCC	2 (13%)	Leiomyosarcoma	1 (13%)	Sebaceous gland carcinoma	2 (25%)
	Glaucoma	1 (11%)	Unspecific eyelid carcinoma	1 (25%)	Retinoblastoma	1 (14%)	BCC	1 (13%)	Atrophic optical nerve, retina, choroid	1 (6.7%)	Malignant schwannoma	1 (13%)	Anophthalmic socket inflammation	1 (13%)
	Limbal SCC	1 (11%)			Spindle cell sarcoma	1 (14%)	Malignant schwannoma	1 (13%)	Lymphoma	1 (6.7%)	Metastasis	1 (13%)	BCC	1 (13%)
	Lymphoma	1 (11%)					Meningeoma	1 (13%)	Malignant astrocytoma	1 (6.7%)	Neurofibrosarcoma	1 (13%)	Melanoma	1 (13%)
	Spindle cell sarcoma	1 (11%)							Pleomorphic lacrimal gland carcinoma	1 (6.7%)	Resection after R1	1 (13%)	SCC	1 (13%)
	Unspecific sarcoma	1 (11%)							Sebaceous gland carcinoma	1 (6.7%)	Sebaceous gland carcinoma	1 (13%)		
									Sweat gland carcinoma	1 (6.7%)				
	Other diagnoses	0	Other diagnoses	0	Other diagnoses	0	Other diagnoses	0	Other diagnoses	0	Other diagnoses	0	Other diagnoses	0
	Sum	9	Sum	4	Sum	7	Sum	8	Sum	15	Sum	8	Sum	8
**Sclera** (*n* = 38)	-		Scleritis	1 (100%)	Filtering bleb revision	1 (33%)	Normal	2 (50%)	Filtering bleb revision	2 (29%)	Normal	6 (60%)	Scleritis	4 (31%)
					Normal	1 (33%)	Scleritis	2 (50%)	Scleritis	2 (29%)	Fistula	1 (10%)	Normal	2 (15%)
					Sclerouveitis	1 (33%)			Foreign body	1 (14%)	Foreign body granuloma	1 (10%)	Scar	2 (15%)
									Foreign body granuloma	1 (14%)	Sclera plaque	1 (10%)	Foreign body	1 (7.7%)
									Normal	1 (14%)	Scleritis	1 (10%)	Foreign body granuloma	1 (7.7%)
													Melanozytic lesion	1 (7.7%)
													Nevus	1 (7.7%)
													Scleral plaque	1 (7.7%)
	Other diagnoses	0	Other diagnoses	0	Other diagnoses	0	Other diagnoses	0	Other diagnoses	0	Other diagnoses	0	Other diagnoses	0
	Sum	0	Sum	1	Sum	3	Sum	4	Sum	7	Sum	10	Sum	13
**Anterior chamber angle** (*n* = 37)	-		-		Sclera and trabecular meshwork	3 (100%)	Sclera and trabecular meshwork	14 (67%)	Sclera and trabecular meshwork	5 (71%)	-		Inflammation	2 (33%)
							Hemosiderin	2 (10%)	Connective tissue	1 (14%)			Connective tissue	1 (17%)
							Iridodialysis	2 (10%)	Pigmented trabecular meshwork	1 (14%)			Normal trabecular meshwork	1 (17%)
							Epithelial downgrowth	1 (4.8%)					Pigmented trabecular meshwork	1 (17%)
							Inflammation (trabecular meshwork)	1 (4.8%)					Trabecular meshwork with vacuoles	1 (17%)
							Sclerosis (trabecular meshwork)	1 (4.8%)						
	Other diagnoses	0	Other diagnoses	0	Other diagnoses	0	Other diagnoses	0	Other diagnoses	0	Other diagnoses	0	Other diagnoses	0
	Sum	0	Sum	0	Sum	3	Sum	21	Sum	7	Sum	0	Sum	6
**Anterior chamber **(*n* = 27)	-		Epithelial downgrowth	1 (100%)	Epithelial downgrowth	4 (80%)	Epithelial downgrowth	7 (100%)	-		Inflammation	3 (50%)	Inflammation	3 (38%)
					Inflammation	1 (20%)					Bleeding	2 (33%)	Epithelial downgrowth	2 (25%)
											Foreign body	1 (17%)	Bleeding	1 (13%)
													Connective tissue with hemosiderin	1 (13%)
													Unknown cells	1 (13%)
	Other diagnoses	0	Other diagnoses	0	Other diagnoses	0	Other diagnoses	0	Other diagnoses	0	Other diagnoses	0	Other diagnoses	0
	Sum	0	Sum	1	Sum	5	Sum	7	Sum	0	Sum	6	Sum	8
**Optic nerve** (*n* = 15)	-		Atrophy	1 (100%)	Glioma	2 (40%)	Normal	2 (50%)	-		Malignant nerve sheath tumour	2 (40%)	-	
					Atrophy	1 (20%)	Glioma	1 (25%)			Meningioma	2 (40%)		
					Normal	1 (20%)	Meningioma	1 (25%)			Atrophy	1 (20%)		
					Postoperative gliosis	1 (20%)								
	Other diagnoses	0	Other diagnoses	0	Other diagnoses	0	Other diagnoses	0	Other diagnoses	0	Other diagnoses	0	Other diagnoses	0
	Sum	0	Sum	1	Sum	5	Sum	4	Sum	0	Sum	5	Sum	0

This table presents the number and relative frequency of diagnoses from most topographical areas received within each 10-year interval (with the interval 2005–2015 spanning 11 years) during the observation period from 1945 to 2015. Diagnoses from the main topographical areas are listed as “Top 10”, while those from less frequent areas are listed as “Top 5”. In cases where diagnoses share the same rank, there may be more than 10 or 5 entries. Discrepancies with the sum values of each interval are categorised as “Other diagnoses”. Excluded from this analysis are 6,276 diagnoses, comprising eyeball (*n* = 3,555), temporal arteries (*n* = 1,517), unspecified specimens (*n* = 601), lens (*n* = 370), non-ophthalmic specimens (*n* = 173), and evisceration (*n* = 60). Note that percentages may not always total 100% due to rounding.

BCC = Basal cell carcinoma

BK w = Bullous keratopathy with previous intraocular surgery

BK w/o = Bullous keratopathy without previous intraocular surgery

CIN = Conjunctival intraepithelial neoplasia

CNV = Choroidal neovascularisation

FH = Fibrous histiocytoma

MFH = Malignant fibrous histiocytoma

Normal = Normal exenteration specimens (details see text)

PHPV = Primary hyperplastic vitreous

PICPS = Pluripotent immune cell proliferative syndrome

Resection after R1 = Secondary surgery after incomplete resection. For exenteration specimens see details in the text.

Salzmann = Salzmann nodular degeneration

SCC = Squamous cell carcinoma

**Table 2:** Changes in median patient age at time of surgery across all topographical regions

**Eyelid**	1945–1954	1955–1964	1965–1974	1975–1984	1985–1994	1995–2004	2005–2015
Chalazion	27 (17–56)	34 (2–72)	43 (1–78)	50 (1–87)	42 (1–87)	45 (0–93)	45 (1–97)
Basal cell carcinoma (BCC)	64 (46–78)	65 (41–85)	63 (20–91)	71 (18–94)	71 (26–100)	71 (21–100)	74 (22–102)
Papilloma	57 (37–75)	61.5 (9–82)	63 (2–100)	63 (3–100)	64 (3–91)	64 (3–96)	64 (4–98)
Resection after R1 (Secondary surgery after incomplete resection)	-	-	-	-	73 (38–90)	71 (21–95)	73 (22–97)
Nevus	31.5 (14–49)	54 (14–73)	46 (7–80)	58 (5–87)	53 (4–85)	53 (4–92)	55 (5–94)
Epidermal cyst	16 (2–52)	5 (2–71)	43 (1–74)	57 (3–87)	58 (2–89)	56.5 (2–87)	62 (1–97)
Xanthelasma	48 (45–48)	53.5 (46–61)	48.5 (36–74)	50 (30–69)	49 (21–79)	53 (20–86)	53 (25–83)
Granuloma	46.5 (22–71)	40 (2–65)	29 (0–76)	41 (2–79)	49 (0–84)	61 (6–91)	56 (5–85)
Dermoid cyst	37 (2–72)	17 (15–59)	5 (0–78)	6 (1–76)	5.5 (0–61)	4 (0–82)	4 (0–90)
Squamous cell carcinoma (SCC)	66 (45–82)	64.5 (43–85)	66.5 (53–77)	78 (67–93)	81 (51–90)	70 (21–94)	75 (23–94)
**Cornea**	1945–1954	1955–1964	1965–1974	1975–1984	1985–1994	1995–2004	2005–2015
Fuchs‘dystrophy	-	-	70 (36–87)	70 (36–100)	72 (50–100)	71(42–90)	72 (27–95)
Keratoconus	-	21 (19–23)	25 (13–63)	27 (11–83)	30 (15–92)	37 (14–79)	41 (14–83)
Inflammation	47.5 (24–68)	52 (22–78)	51 (2–83)	47 (0–82)	54 (0–85)	60 (2–95)	59 (1–92)
Transplant failure	36.5 (23–58)	30 (22–82)	41 (7–82)	58 (17–81)	67 (22–94)	62 (17–84)	63 (3–90)
Scar	20^a^	33 (18–54)	43.5 (3–85)	49 (1–88)	61 (12–86)	56 (17–92)	61 (1–95)
Bullous keratopathy with previous intraocular surgery (BK w)	-	-	60.5 (54–67)	66 (22–83)	76 (41–91)	77 (53–88)	75 (8–92)
Bullous keratopathy without previous intraocular surgery (BK w/o)	-	-	71 (64–75)	62.5 (23–87)	71.5 (17–86)	70 (7–92)	71 (12–100)
Ulcer	74.5 (72–77)	57.5 (15–78)	51 (16–63)	66 (18–89)	60 (0–85)	67 (1–93)	70.5 (5–99)
Infection	-	56 (41–67)	61.5 (40–74)	31 (12–72)	27 (26–85)	50 (15–95)	58 (1–89)
Squamous cell carcinoma	73.5 (57–77)	75^a^	49 (29–69)	-	-	74^a^	-
**Conjunctiva**	1945–1954	1955–1964	1965–1974	1975–1984	1985–1994	1995–2004	2005–2015
Pterygium	-	36 (31–41)	49 (21–71)	49 (30–89)	55 (11–85)	54 (25–90)	61 (4–96)
Nevus	48 (27–52)	19 (8–66)	22.5 (6–64)	29 (0–81)	31 (3–89)	31 (2–93)	34 (3–89)
Pyogenic granuloma	8 (4–12)	17 (7–77)	38 (29–68)	41.5 (1–85)	50 (6–81)	48.5 (3–93)	56 (2–92)
Conjunctivitis	53.5 (17–75)	47 (6–63)	50 (5–83)	43.5 (7–77)	52.5 (7–91)	48 (9–89)	54 (0–91)
Conjunctival intraepithelial neoplasia (CIN)	-	84.5 (83–86)	72 (60–93)	69 (35–79)	68 (20–81)	65 (22–90)	65 (27–91)
Papilloma	31 (22–58)	50 (28–72)	40 (4–70)	41 (6–88)	39 (16–84)	53 (3–93)	54 (3–88)
Cyst	28 (14–42)	23 (9–70)	37 (2–72)	48 (1–78)	54.5 (7–78)	58 (4–92)	60 (1–85)
Pinguecula	50.5 (45–56)	59.5 (38–81)	38.5 (27–68)	47.5 (34–62)	56 (26–80)	51.5 (18–82)	53 (26–93)
Granuloma	27 (13–45)	20 (10–66)	10 (1–53)	44 (3–76)	33 (21–78)	56 (4–94)	50 (11–78)
Squamous cell carcinoma	62 (53–78)	43 (5–76)	76 (57–95)	70 (50–84)	59 (56–61)	56 (28–91)	57 (34–91)
**Orbit**	1945–1954	1955–1964	1965–1974	1975–1984	1985–1994	1995–2004	2005–2015
Inflammation	59.5 (55–64)	56 (7–78)	64.5 (18–69)	59 (15–75)	58.5 (6–79)	51 (41–81)	46 (37–79)
Lymphoma	73	-	-	71 (68–74)	66	69 (51–85)	67 (30–87)
Fat tissue	-	14	-	-	66 (55–71)	64 (15–84)	70 (56–80)
Hemangioma	-^b^	51 (48–54)	52 (36–64)	50 (29–59)	58.5 (39–81)	55 (34–80)	47 (45–49)
Metastasis	45	59	56 (40–58)	55.5 (41–70)	67 (37–72)	63.5 (52–80)	71 (62–75)
Granuloma	31	34 (14–54)	47.5 (47–48)	39 (14–64)	50 (17–64)	39 (35–47)	37 (25–60)
Normal Muscle	-	20 (5–66)	48 (6–68)	-	59.5 (3–71)	57 (6–83)	-
Scar	-	30 (26–34)	-	15.5 (14–17)	55.5 (39–72)	-	62.5 (38–87)
Dermoid cyst	0	8	37 (34–40)	38 (6–43)	30 (29–33)	20 (2–39)	-
Melanoma	-	-	50 (33–67)	57 (53–84)	34 (34–34)	60 (53–67)	70 (45–74)
**Lacrimal duct system**	1945–1954	1955–1964	1965–1974	1975–1984	1985–1994	1995–2004	2005–2015
Dacryocystitis	48 (5–76)	50 (0–77)	30 (1–78)	53 (18–87)	63.5 (38–85)	62 (24–93)	73 (17–95)
Canaliculitis	-	-	66.5 (61–72)	62 (27–83)	53 (39–82)	62.5 (47–82)	54.5 (35–87)
Lacrimal sac concrement	-	62	41 (25–57)	44 (28–61)	36 (26–68)	53 (29–80)	51.5 (31–79)
Scar	55	-	27	58 (43–73)	52 (38–83)	76	70
Normal	34	-	-	44 (25–63)	63 (40–86)	52 (17–81)	74 (40–84)
**Uvea**	1945–1954	1955–1964	1965–1974	1975–1984	1985–1994	1995–2004	2005–2015
Iris melanoma	51	60.5 (48–73)	59 (30–74)	67 (32–71)	36 (13–59)	57 (34–79)	72 (16–80)
Normal iris	56	10	53 (32–67)	58 (27–82)	70 (64–84)	59 (5–92)	33 (2–71)
Iritis	51 (40–70)	57 (50–64)	47.5 (2–72)	51.5 (44–59)	-	60.5 (29–81)	55 (21–81)
Iris nevus	42 (29–55)	-	-	36 (1–65)	50 (43–57)	66.5 (28–80)	52 (31–73)
Ciliary body melanoma	-	-	-	-	44 (44)	75 (47–76)	70 (66–74)
**Intraocular tissue**	1945–1954	1955–1964	1965–1974	1975–1984	1985–1994	1995–2004	2005–2015
Trauma-associated without retina	-	-	17.5 (4–31)	23	40.5 (9–91)	52 (2–92)	63.5 (5–97)
Trauma-associated with retina	-	-	-	-	60 (19–88)	61 (5–94)	72 (30–93)
Endophthalmitis	-	-	-	-	-	50	97
Fibrin	-	-	-	-	-	-	73
**Vitreous**	1945–1954	1955–1964	1965–1974	1975–1984	1985–1994	1995–2004	2005–2015
Vitritis	-	-	-	32.5 (27–38)	58.5 (0–87)	63 (8–90)	73 (11–89)
Normal	-	-	38	16	63.5 (60–67)	45 (24–83)	70 (40–79)
Lymphoma	-	-	-	-	58	75.5 (62–79)	78 (63–83)
Bleeding	-	-	-	54	47 (5–70)	54 (37–86)	67 (65–79)
Persistent hyperplastic primary vitreous (PHPV)	-	-	-	1	13.5 (0–27)	0 (0–1)	14
**Lacrimal gland**	1945–1954	1955–1964	1965–1974	1975–1984	1985–1994	1995–2004	2005–2015
Dacryoadenitis	-	-	55	54 (14–70)	42 (41–64)	48 (2–74)	53 (42–64)
Normal	-	17.5 (16–19)	-	73	68 (42–72)	47 (28–51)	37 (4–78)
Pleomorphic adenoma	-	-	36 (26–55)	45 (37–58)	46.5 (29–67)	70 (32–87)	53.5 (40–67)
Cyst	40.5 (40–41)	20	52	54 (41–61)	64	20.5 (2–39)	46 (29–69)
Hypertrophy	-	-	-	-	65	-	-
**Retina**	1945–1954	1955–1964	1965–1974	1975–1984	1985–1994	1995–2004	2005–2015
Choroidal neovascularisation	-	-	-	-	-	74 (44–84)	81 (13–90)
Epiretinal gliosis	-	-	-	-	71	47.5 (39–64)	74 (23–82)
Proliferative vitreoretinopathy	-	-	-	-	55 (53–75)	75 (22–89)	51.5 (17–84)
Connective tissue	-	-	-	22	-	83	65
Normal	-	-	41	-	83	77	29 (16–67)
**Exenteration**	1945–1954	1955–1964	1965–1974	1975–1984	1985–1994	1995–2004	2005–2015
Basal cell carcinoma	44	-	-	61	72 (52–81)	-	93
Squamous cell carcinoma	48	66.5	-	-	56.5 (44–69)	-	84
Inflammation	-	64	48 (24–72)	-	-	-	45
Normal	-	-	-	70 (69–71)	-	69 (11–67)	-
Lymphoma	11	-	62 (9–62)	-	80	-	-
**Sclera**	1945–1954	1955–1964	1965–1974	1975–1984	1985–1994	1995–2004	2005–2015
Normal	-	-	65	50.5 (44–57)	62	71 (42–81)	61 (56–66)
Scleritis	-	54	-	51 (28–74)	32.5 (10–55)	61	53 (32–77)
Filtering bleb revision	-	-	76	-	68 (57–79)	-	-
Foreign body granuloma	-	-	-	-	23	71	30
Foreign body	-	-	-	-	77	-	65
Sclerouveitis	-	-	20	-	-	-	-
**Anterior chamber angle**	1945–1954	1955–1964	1965–1974	1975–1984	1985–1994	1995–2004	2005–2015
Sclera and trabecular meshwork	-	-	65 (62–74)	58 (9–68)	48 (15–82)	-	-
Inflammation	-	-	-	53	-	-	72.5 (72–73)
Connective tissue	-	-	-	-	48	-	66
Hemosiderin	-	-	-	53.5 (40–67)	-	-	-
Pigmented trabecular meshwork	-	-	-	-	62	-	71
Iridodialysis	-	-	-	56.5 (49–64)	-	-	-
**Anterior chamber**	1945–1954	1955–1964	1965–1974	1975–1984	1985–1994	1995–2004	2005–2015
Epithelial downgrowth	-	28	64.5 (51–79)	65 (40–81)	-	-	55.5 (38–73)
Inflammation	-	-	46	-	-	51 (44–52)	83 (70–84)
Bleeding	-	-	-	-	-	75.5 (65–86)	42
Foreign body	-	-	-	-	-	33	-
Unspecific cells	-	-	-	-	-	-	72
**Optic nerve**	1945–1954	1955–1964	1965–1974	1975–1984	1985–1994	1995–2004	2005–2015
Meningioma	-	-	-	61	-	26 (15–37)	-
Glioma	-	-	17 (5–29)	8	-	-	-
Atrophy	-	62	29	-	-	5	-
Normal	-	-	57	35 (34–36)	-	-	-
Malignant nerve sheath tumour	-	-	-	-	-	74.5 (67–82)	-

This table presents the median patient age and age range at the time of surgery for each 10-year interval (with the interval 2005–2015 spanning 11 years) for frequently submitted diagnoses throughout the entire observation period from 1945 to 2015. The diagnoses are ordered by listing the most frequent diagnoses for each topographic region, arranged in descending order based on the mean values of their interval-based percentages.

^a^ Age information is available for only one patient

^b^ Age information not available

### Eyelid

Throughout the observation period, the eyelid emerged as the most common topographical site, with a total of 21,764 diagnoses assessed. Among these, chalazion accounted for the highest proportion of eyelid diagnoses at 18%, followed by basal cell carcinoma (BCC) at 16%, papilloma at 16%, secondary surgery due to incomplete (R1) resection at 6.5%, and nevus at 4.6%.

In 1946, there was just one case of chalazion, accounting for 6.7% of the 15 eyelid diagnoses. Over the years, the annual number of chalazion cases steadily increased, reaching 256 cases, or 25% of the 1,043 eyelid diagnoses, by 2015 (Fig. 1). Similarly, the first diagnoses of BCC were recorded in 1946, with four cases making up 27% of that year’s diagnoses. This number grew continuously, peaking at 201 cases, or 20% of the 995 eyelid diagnoses, in 2014.

Throughout our observation period, there was also a steady increase in the annual numbers of other conditions: papilloma cases rose from two (13%) in 1946 to 152 (15%) in 2015; resections following incomplete (R1) excision increased from one case (0.3%) out of 331 eyelid diagnoses in 1990 to 129 cases (13%) in 2014; and nevi cases grew from two (11%) out of 19 eyelid diagnoses in 1954 to 50 cases (4.7%) in 2014.

A visual inspection shows that the frequency distributions of the five major eyelid diagnoses, categorized by patient age at the time of surgery, each deviate from a normal distribution (Fig. 5). For BCC, papilloma, and resections following incomplete (R1) excision, the distributions exhibit a left-skewed asymmetry. The peak ages for diagnoses, indicated by mode values, are 73 for BCC (with 139 cases), 66 for papilloma (with 103 cases), and 80 for resections following R1 (with 50 cases). The highest ages of patients undergoing BCC surgery were 102 years (with one case), 100 years (with three cases), and 98 years (with two cases). Furthermore, two papilloma surgeries were conducted on patients aged 100 years and one resection following incomplete (R1) excision on a patient aged 97 years. Regarding chalazia, the age distribution starts below 6 months (with six cases), peaks at age 4 (with 15 cases), and then declines to six cases by age 10. There is a notable increase in cases starting from age 12 to 17, with a total of 63 cases. This upward trend continues until age 46, peaking at 94 cases, after which there is a decline, with only one case recorded at age 93; the highest age at surgery being 97 years. The age distribution for nevi is the least notable, peaking at 28 diagnoses at age 59, with the highest age at surgery being 94 years (with one case).

Upon examining individual intervals, *chalazia* accounted for a smaller relative proportion of all eyelid diagnoses up until the period from 1975 to 1984, with a relative frequency ranging between 5.5% and 8.4% in each interval (Table 1). Subsequently, their frequency increased, ultimately becoming the primary eyelid diagnosis during the last two analyzed intervals. This trend peaked at 22% (*n* = 2,293) in the period from 2005 to 2015, with a notable high of 27% (*n* = 241) in 2010. The median patient age was at its lowest, 27 years, during the interval from 1945 to 1954, and reached its highest, 50 years, in the period from 1975 to 1984. Additionally, the range of patient ages (age_max_ - age_min_) expanded significantly over the course of the study, increasing from 39 years (17–56) to 96 years (1–97) (Table 2).

*BCC* consistently ranked among the “Top 3” eyelid diagnoses throughout the study period, with relative frequencies ranging from 15% to 26%. The highest percentage was observed in the interval from 1965 to 1974, during which BCC accounted for 26% of diagnoses (*n* = 196), peaking at 37% in 1964. However, this percentage subsequently declined to 15% (*n* = 1,541) in the period from 2005 to 2015 (Table 1). The median age of patients undergoing surgical removal of periocular BCC was lowest during the first three analyzed intervals, ranging between 63 and 65 years. This increased to 71 years during the subsequent three intervals and further to 74 years in the period from 2005 to 2015 (Table 2). Additionally, the range of patient ages more than doubled, expanding from 32 years (46–78) in the first interval to 80 years (22–102) in the seventh interval.

*Papillomas* consistently ranked within the “Top 3” diagnoses during each interval, starting with 12 cases (14%) in the period from 1945 to 1954. Their prevalence varied between 26% (*n* = 196) in 1975–1984 and 11% (*n* = 1,188) in 2005–2015, with a peak contribution of 48% (*n* = 13) in 1956 (Table 1). The median patient age exhibited a slight increase over the observation period, rising from 57 years in 1945–1954 to 64 years in 1985–1994, where it plateaued (Table 2). Additionally, a considerable widening of age ranges was observed, expanding from 38 years (37–75) in 1945–1954 to 94 years (4–98) in the final interval. Since 1965, papillomas have been subclassified histologically. During the interval from 1975 to 1984, seborrheic keratosis and squamous papilloma accounted for 73 and 14% of cases, respectively. This distribution shifted to 67 and 27% in 1985–1994, 53 and 39% in 1995–2004, and 42 and 58% in 2005–2015.

Instances of *secondary surgery following incomplete (R1) resection* have been documented since 1990. The rates of tumour-free resections in secondary surgeries after incomplete resection were 77% from 1985 to 1994, 86% from 1995 to 2004, and 95% between 2005 and 2015 (data not shown). The median age of patients at the time of surgery ranged from 71 to 73 years, with the age range expanding from 52 years (38–90) to 75 years (22–97) (Table 2).

*Nevi* accounted for 2.4% (*n* = 2) of total eyelid diagnoses from 1945 to 1954, rising to 7.8% (*n* = 58) between 1965 and 1974, before decreasing to 3.3% (*n* = 349) from 2005 to 2015 (Table 1). The median age of patients at the time of surgery increased from 31.5 years in 1945–1954 to 58 years in 1975–1984, followed by a slight decline to 55 years in 2005–2015. Meanwhile, the age range expanded significantly from 35 years (14–49) to 89 years (5–94) (Table 2).

Interestingly, although *squamous cell carcinoma* (SCC) did not rank among the five most common eyelid diagnoses throughout our study period, it was most prevalent during the first two intervals (1945–1964), accounting for 22% (*n* = 18) and 12% (*n* = 25) of cases (Table 1). However, its incidence subsequently fell below 2% in the remaining intervals. The median age of patients at the time of surgery rose from 66 years to 81 years between 1985 and 1994, followed by a slight decrease to 75 years from 2005 to 2015. Meanwhile, the age range nearly doubled, expanding from 37 years (45–82) in 1945–1954 to 71 years (23–94) in 2005–2015 (Table 2).

Throughout the period from 1945 to 2015, *epidermal cysts* and *dermoid cysts* exhibited a similar frequency of occurrence, with rates of 2.4–6.3% and up to 4.8%, respectively. Concerning the median age at surgery for dermoid cysts, a decline was observed from 37 years in 1945–1954 to 17 years between 1955 and 1964. This trend continued, with the median age further decreasing to 6 years and eventually to 4 years in subsequent intervals (Table 2). Additionally, the age range broadened from 70 years (2–72) to 90 years (0–90). In contrast, for epidermal cysts, the median age at surgery increased from 16 years to 62 years, accompanied by a significant expansion in the age range from 50 years (2–52) to 96 years (1–97).

Although not among the “Top 5” diagnoses as well, *xanthelasma* was relatively frequent within the first interval (*n* = 5, 6.0%, 1945–1954). Its relative frequency subsequently dropped below 1% (*n* = 8, 1955–1964), before rising again to 1.6% (*n* = 78) and eventually to 2.9% (*n* = 305) in the last two intervals (1995–2015). The median age at surgery for xanthelasma remained relatively stable, while the age range widened from 3 years (45–48) in 1945–1954 to 58 years (25–83) in the final interval.

### Cornea

The cornea emerged as the second most frequent topographical site in our database, with a total of 7,319 corneal diagnoses examined in this study. It is important to note that corneal diagnoses from enucleated or eviscerated eyes were excluded from this analysis. Throughout the study period, the most prevalent diagnosis was Fuchs’ dystrophy, accounting for 19% of cases. This was followed by keratoconus at 13%, keratitis (unspecified) at 11%, transplant failure at 11%, and scar tissue at 10%.

Fuchs’ dystrophy was first diagnosed in 1966, with one case accounting for 14% of the seven corneal diagnoses that year. The annual number of cases increased significantly to 267, representing 50% of the 536 total corneal diagnoses in 2015 (Fig. 2). Following the initial diagnosis of keratoconus in 1960 (*n* = 1, 27%), there was a continuous yet moderate rise to 66 cases, or 21% of the 313 total corneal diagnoses, by 2008. However, by 2015, the number had decreased to 35 cases, comprising just 6.5%. Throughout our observation period, the annual number of keratitis diagnoses increased from three cases (43%) out of seven in 1945 to 47 cases (20%) out of 233 corneal diagnoses in 2004. Similarly, transplant failures rose from one case (14%) in 1945 to 71 cases (27%) in 2010, eventually reaching 66 cases (12%) in 2015. Lastly, diagnoses of scar tissue increased from one case (25%) out of four corneal diagnoses in 1951 to 45 cases (11%) out of 423 in 2014.

The age distribution for Fuchs’ dystrophy is notably symmetrical, showing a mode value of 74 years (*n* = 74). The age at surgery ranges from 27 years (with one case) to 100 years (with two cases). Visual inspection suggests that the frequency distributions of the other four major corneal diagnoses by patient age deviate from a normal distribution (Fig. 6). The age distribution for keratoconus exhibits a right-skewed asymmetry, with diagnoses occurring from age 13 onwards and a modal value of 28 years (*n* = 34). The highest age at surgery for keratoconus was 92 years (*n* = 1). In contrast, the age distribution curves for keratitis, transplant failure, and scar tissue are broad and flat. These curves do not exceed 31 keratitis diagnoses at age 78, 22 transplant failure diagnoses at ages 65 and 73, and 19 scar tissue diagnoses at age 68. It is noteworthy that three cases of keratitis were diagnosed in patients under six months of age. Subsequently, the number of cases varies between one and three annually up to the age of 10 years. The highest patient ages at surgery were 95 years (*n* = 1) for keratitis, 94 years (*n* = 1) for transplant failure, and again 95 years (*n* = 1) for scar tissue.

Regarding individual intervals, no cases of *Fuchs’ dystrophy* were recorded until 1966. Subsequently, the number of cases gradually increased from 21 in the period 1965–1974 to 62 in 1985–1994, and further to 150 in 1995–2004 (Table 1). The most significant rise occurred in the last interval, 2005–2015, with a 7.2-fold increase to 1,082 cases. Notably, 57% of all samples with Fuchs’ dystrophy were submitted during the final five years of this period (2011–2015). The relative frequency of Fuchs’ dystrophy also climbed from 7.6% in 1985–1994 to 25% in 2005–2015, reaching a peak of 50% in 2015. The median age showed a slight increase from 70 years in 1965–1974 to 72 years in 2005–2015 (Table 2). The age range varied, from 48 years (42–90) in 1995–2004 to 64 years (36–100) in 1975–1984, without indicating any specific trend.

*Keratoconus* was initially diagnosed with two cases in the period 1955–1964. This number rose to 33 cases (13%) in 1965–1974, further increasing to 122 cases (22%) in 1975–1984, and then to 155 cases (19%) in 1985–1994. By 1995–2004, the count had reached 217 cases (18%). This trend established keratoconus as the most common corneal diagnosis between 1975 and 2004, with a peak contribution of 30% (*n* = 17) in 1980 (Table 1). However, the percentage of keratoconus samples subsequently declined to 10% in the period 2005–2015. There was a continuous increase in the median age, rising from 21 years in 1955–1964 to 41 years in 2005–2015 (Table 2). The age range varied, starting at 4 years (19–23) in 1955–1964, increasing to 77 years (15–92) in 1985–1994, and finally decreasing to 69 years (14–83).

*Keratitis* emerged as one of the two primary corneal diagnoses during the first two intervals and again within the fourth and sixth intervals evaluated. Included are, among others, bacterial and viral infections (pathogens histologically not identified), and acute and chronical inflammation of various origin. Keratitis accounted for 22% (*n* = 6), 27% (*n* = 13), 19% (*n* = 107), and 14% (*n* = 165) of cases, respectively. The median age of patients at the time of surgery varied from 47.5 years in the period 1945–1954 to 60 years in 1995–2005 with an age range spanning from 2 to 95 years. Notably, the differences in age range considerably increased across our study period from 44 to 93 years. During the interval from 1945 to 1954, the youngest patient undergoing treatment was 24 years old. By the end of our observation period, the youngest patient age had decreased to just 1 year.

Following a relatively modest increase from four cases (15%) in the period 1945–1954 to 65 cases (5.3%) in 1995–2004, the absolute number of *failed transplants* surged 9.3-fold to 603 cases (14%). This placed transplant failure in second position in the ranking of corneal diagnoses, following Fuchs’ dystrophy, during the last interval. Notably, these cases comprised 75% of all transplant failures recorded between 1945 and 2015 (Table 1). The median age of patients diagnosed with transplant failure was in their thirties during the first two intervals (1945–1964) and rose to their sixties during the last three intervals (1985–2015) (Table 2). Additionally, the differences in age range expanded from 35 years (23–58) to 63 years (3–90) over the observation period.

The relative frequency of *scar tissue* samples rose from 7.4% (*n* = 2) in the period 1945–1954 and 14% (*n* = 7) in 1955–1964 to a considerable 29% (*n* = 76) in 1965–1974. Despite a steady increase in their numbers, their proportion subsequently declined to 16% (*n* = 89) in 1975–1984 and further to 8.4% (*n* = 368) in 2005–2015. Nevertheless, half of all corneal lesions diagnosed with scarring (50%, *n* = 368) were excised within the last interval analyzed. We observed an increase in the median patient age from 20 years (1945–1954) to 61 years (2005–2015), with a remarkable age range difference of 94 years (1–95).

Although *bullous keratopathies* (BK) were not among the “Top 5” corneal diagnoses during our study period, they warrant mention due to their notable contribution during the sixth and seventh time intervals. BK diagnoses were categorized into two groups: those with a history of intraocular surgery (“BK w”) and those without (“BK w/o”). The initial four “BK w” diagnoses were recorded in the period 1965–1974. This was followed by 38 BK diagnoses in 1975–1984, of which 29 were “BK w” and 9 were “BK w/o”. Up until 1985–1994, bullous keratopathies associated with intraocular surgery clearly predominated over those without surgical history, with a ratio of 3.2:1. However, this ratio subsequently reversed. In the period 1995–2004, we identified 39 “BK w” cases (3.0%) and 128 “BK w/o” cases (11%), indicating a ratio of 1:3.3. This ratio remained nearly consistent (1:3.6) in 2005–2015, with 79 “BK w” diagnoses (1.8%) compared to 288 “BK w/o” diagnoses (6.6%) within the final interval.

The median age of patients diagnosed with “BK w” rose from 60.5 years in the period 1965–1974 to 77 years in 1995–2004. In contrast, the median age for “BK w/o” exhibited less fluctuation, ranging from 62.5 to 71.5 years. Additionally, the age range for “BK w” widened significantly, expanding from 13 years (54–67) in 1965–1974 to 84 years (8–92) in 2005–2015. Similarly, for “BK w/o”, the age range broadened from 11 years (64–75) to 88 years (12–100) over the same period. For further details on these findings, please refer to our previous paper [20].

### Conjunctiva

Specimens from the conjunctiva, including the caruncle, were the third most common topographical site, with 5,963 conjunctival diagnoses archived in our laboratory. Pterygium accounted for 29% of all excised conjunctival lesions and nevus for 12%, followed by pyogenic granuloma (7.0%), conjunctivitis (6.2%) and conjunctival intraepithelial neoplasia (CIN) (5.0%).

The first case of pterygium was diagnosed in 1959, being 6.7% of 15 conjunctival diagnoses in total, their annual numbers increasing to 127 (41%) (Fig. 3) of 309 conjunctival diagnoses in 2015. Nevi were first found in 1947 (*n* = 2, 50%), their number rose to 35 (11%) in 2015. Annual diagnoses of pyogenic granuloma increased from one (14%) of 7 conjunctival diagnoses in 1949 to 28 (9.6%) of 292 diagnoses in 2014, conjunctivitis from one (25%) in 1946 to 25 (12%) of 203 conjunctival diagnoses in 2002, finally to 21 (6.8%) in 2015, and CIN from 1 (11%) of 9 conjunctival diagnoses in 1960 to 27 (10%) of 261 in 2012 and 25 (8.1%) in 2015.

The frequency distributions of the five major conjunctival diagnoses by patient age clearly deviate from the shape of a normal distribution (Fig. 7). For pterygium, there is a significant rise starting beyond age 24, peaking at age 46 (with 49 cases). This is followed by a plateau-like section with a ‘staccato’ pattern, which then sharply descends from age 76 (with 46 cases). The highest age at surgery was found to be 96 years (with one case). The age distribution for nevi exhibits an asymmetric, right-skewed shape, rising sharply beyond age 5 and peaking at age 14 (with 23 cases). The highest age at surgery for nevi was 93 years (with one case). Pyogenic granuloma, conjunctivitis, and CIN each display broad and flat age distributions. These distributions do not exceed 12 diagnoses of pyogenic granuloma at age 65, 10 diagnoses of conjunctivitis at ages 47 and 53, and 15 diagnoses of CIN at age 69. The youngest age at surgery for CIN was 20 years (one case). The highest patient ages at surgery were 93 years (with one case) for pyogenic granuloma, 91 years (with two cases) for conjunctivitis, and 91 years (with one case) for CIN.

As regards individual intervals, while there was no case of *pterygium* registered in interval 1945–1954 and only two cases (2.4%) were found in 1955–1964, numbers rose sharply with a 6.5-fold increase from 20 cases in 1975–1984 to 130 in 1985–1994 (Table 1), which has since become the most common conjunctival diagnosis. Most pterygia (97%) were excised during the last three intervals, with 61% of all cases diagnosed between 1945 and 2015 occurring in 2005–2015. The median age rose from 36 years in 1955–1964 to 61 years in 2005–2015 (Table 2). Age range differences increased dramatically from 10 years (31–41) in 1955–1964 to 92 years (4–96) in 2005–2015.

*Conjunctival nevi* were submitted for examination throughout the entire analysed period. Starting with only five cases each in the first two intervals (10% and 6.0%, respectively), absolute numbers increased 9-fold to 46 cases (16%) in 1965–1974 and doubled again to 85 (20%) in 1975–1984. Nevus was the most common diagnosis during those two intervals, whereby absolute numbers continued to rise, although rather slightly, to 99 cases (16%) in 1985–1994, and to 165 and 281 (10% each) in 1995–2004 and 2005–2015. The median age at the time of excision was 48 years in 1945–1954, then decreased considerably to 19 years in 1955–1964 from where it increased to just 34 years in 2005–2015. During the entire observational period, nevi were excised most often at a patient’s age of 14 years (Fig. 7). The range of patient ages widened from 25 years (27–52) in 1945–1954 to 91 years (2–93) in 1995–2004, followed by 86 years (3–89) in 2005–2015 (Table 2).

The relative frequency of *pyogenic granulomas* decreased from 4.2% (*n* = 2) and 3.6% (*n* = 3) in the first two intervals (1945–1964) to their lowest occurrence in 1965–1974 (2.4%, *n* = 7), then climbing to their highest percentage in 1985–1994 (11%, *n* = 71) (Table 1). 70% of all pyogenic granulomas were removed during the last two intervals (1995–2015). The median age increased remarkably from just 8 and 17 years, respectively, during the first two intervals to 56 years in 2005–2015, with age range differences once again changing dramatically from just 8 years (4–12) to 90 years (2–92) across the entire study period (Table 2). An almost comparable increase in patient’s median age was noted in conjunctival cysts and in granulomas, from 28 (27) years in 1945–1954 to 60 (56) years in 2005–2015. Both conjunctival cysts and granulomas showed a widening of the range of patient ages at surgery as well, from 28 years (14–42) and 32 years (13–45) in 1945–1954 to 84 years (1–85) in 2005–2015 and 90 years (4–94) in 1995–2004, respectively.

*Conjunctivitis* was the primary reason for the histopathological examination of conjunctival tissue from 1945 to 1964, accounting for 21% (*n* = 10) and 20% (*n* = 17) of cases (Table 1). Further histological subclassification of conjunctivitis specimens, although not shown, revealed that chronic disease of unknown origin constituted the majority of cases. Vernal conjunctivitis was diagnosed three times in the interval 1965–1974 and twice in 1975–1984. Graft-versus-host disease, as a diagnosis, was documented only during the last two intervals, with 34 cases in 1995–2004 and 6 cases in 2005–2015. Although the median age of patients at the time of surgery ranged between 43.5 and 54 years throughout our observation period (Table 2), the age range expanded from 58 years (17–75) in 1945–1954 to 91 years (0–91) in 2005–2015.

The first three *CIN* diagnoses (3.6%) were made in 1955–1964; their number increased slightly over the study period, with percentages between 3.1% and 5.3%, and finally reached 188 (6.6%) in 2005–2015 (Table 1). While patient ages ranged from 83 to 86 years with a median of 84.5 years in 1955–1964, this parameter rose only slightly to 86 years within 1995–2015 (Table 2). However, the range differences increased to 68 years (22–90) in 1995–2004 and 64 years (27–91) in our last interval.

### Orbit

This analysis included 535 orbital diagnoses, making the orbit the sixth most common anatomical site (1.2%). While the eyeball (8.2%) and temporal artery (3.5%) ranked as the fourth and fifth most frequent locations, respectively, they had already been addressed elsewhere [20]. Lesions of the lacrimal gland, the optic nerve, and cases involving exenteration were assessed separately. The most frequent orbital diagnosis was inflammation, accounting for 12% of cases, followed by lymphoma at 9.5%, fat tissue (prolapse) at 6.7%, metastasis at 6.5% and hemangioma at 6.5%. While the number of cases from primary topographical sites—such as the eyelid, cornea, and conjunctiva—saw substantial increases over our observation period (127-fold, 162-fold, and 59-fold, respectively), the rise in orbital specimens was comparatively modest at 6-fold.

A visual analysis of the frequency distributions of the five primary orbital diagnoses by patient age reveals a slight increase in cases for those over the age of 35. The age distribution curves for metastasis and hemangioma peak at age 59, with four cases each. For orbital inflammation and fat tissue removal, the peaks occur at ages 46 and 56, respectively, with three cases each. Notably, patients diagnosed with orbital lymphoma have a modal age of 77, with seven cases (Fig. 8). Conversely, the frequency of most other diagnoses tends to decline beyond the age of 75.

*Orbital inflammation* emerged as the primary histological diagnosis during the intervals 1945–1954, 1955–1964, and 1975–1984, with relative frequencies of 14% (*n* = 2), 42% (*n* = 14), and 13% (*n* = 12), respectively (Table 1, Fig. 4). Histologically, inflammation has been subclassified into pseudotumour, myositis, and inflamed scar tissue. Orbital pseudotumour was explicitly documented in varying numbers across several intervals: five cases in 1955–1964, three cases in 1965–1974, nine cases in 1975–1984, seven cases in 1985–1994, 12 cases in 1995–2004, and seven cases in 2005–2015. Myositis, on the other hand, was diagnosed three times in 1965–1974, twice in 1975–1984, and once each in the intervals 1955–1964, 1985–1994, and 1995–2004. Inflamed scar tissue was diagnosed three times during both the intervals 1955–1964 and 1985–1994, and once in 1975–1984. The median age at diagnosis for orbital inflammation showed a marked decrease over time, dropping from 59.5 years (*n* = 2) in 1945–1954 to 46 years (*n* = 8) in 2005–2015 (Table 2).

Until 1994, only 3 *lymphoma* cases were recorded in our series: one each in 1976, 1978, and 1989. However, there was a noticeable increase to 24 cases in each of the last two intervals, making lymphoma the most common orbital diagnosis during those periods (Table 1). During the two most recent study intervals (1995–2004 and 2005–2015), lymphomas represented 17% and 28% of all orbital specimens, respectively. For orbital lymphoma, the patients’ age ranged from 68 to 74 years in the interval 1975–1984 and from 30 to 87 years in 2005–2015 (Table 2).

The first histological examination of prolapsed *orbital fat* at our ophthalmic pathology laboratory dates back to 1955 (Table 1). Between 1985 and 1994, 9 such specimens were recorded, accounting for 8.7% of all orbital diagnoses. This condition rose to become the third most common orbital diagnosis from 1995 to 2004 (*n* = 15, 10%) and subsequently the second most frequent in our last study interval (*n* = 11, 13%). The median age of patients at the time of prolapsed fat or metastatic lesion removal showed little variation across the last three study periods, consistently falling between 64 and 71 years. In contrast, patients undergoing hemangioma surgery were notably younger, with a median age ranging from 47 to 59 years (Table 2).

*Metastasis* featured among the ten most common orbital diagnoses in nearly all study intervals, with proportions ranging from 4.6% to 7.1% (Table 1). Two exceptions were noted: only one specimen was examined in both 1975 and 1983, and during the 1995–2004 period, metastasis accounted for 17 diagnoses, ranking as the second most frequent orbital pathology with a relative frequency of 12%.

Throughout the entire study period (1945–2015), *hemangioma* remained one of the eight most frequent orbital diagnoses (Table 1). Its relative frequency peaked at 10% (*n* = 9) of all orbital diagnoses between 1975 and 1984, but declined to just 2.4% (*n* = 2) in the final study interval.

### Lacrimal duct system

The lacrimal duct system presented with 303 histopathological diagnoses, among which *dacryocystitis* was the most prevalent, accounting for 53% of cases overall and emerging as the leading diagnosis in each interval (Table 1). The relative frequencies varied, starting at 72% (*n* = 23) in 1945–1954 and ascending to a peak of 89% (*n* = 42) in 1955–1964, marking it as the most frequent diagnosis. Subsequently, there was a decline to 40% (*n* = 27) in the interval 2005–2015. The majority of dacryocystitis cases were characterized by chronic inflammation, with two cases attributed to tuberculosis infection, occurring once in each of the first two intervals (not shown in Table 1). The median age of patients was 48 years during the interval 1945–1954 and 50 years in 1955–1964. This figure then decreased to 30 years in 1965–1974, followed by an increase to 73 years in the most recent interval.

*Canaliculitis*, accounting for 14% of cases, was the next most frequent diagnosis, identified through histopathological findings of canalicular concretions. Although no cases of canaliculitis were recorded in the first two intervals, its relative frequency varied between 14% (*n* = 3) in 1965–1974 and 30% (*n* = 20) in 2005–2015. (Table 1). During the same time span, the patients’ median age decreased from 66.5 years to 54.5 years (Table 2).

*Concrements* *in the* *lacrimal sac* were detected almost as frequently, constituting 13% of all lacrimal duct diagnoses. The first instance of such a diagnosis, accounting for 2.1%, was recorded in the interval 1955–1964 (Table 1). In 1975–1984, six diagnoses were made, representing 11% of cases. This figure rose to 17 diagnoses in 1995–2004, making it the second most common finding in the lacrimal duct at 24%. Subsequently, in 2005–2015, the relative frequency was 15% (*n* = 10).

### Uvea

Our database recorded 220 diagnoses of uveal samples, encompassing the iris, ciliary body, and choroid. Diagnoses from enucleated eyeballs are not included. The most common diagnoses, in terms of all excised uveal specimens, were normal iris and iris melanoma, each accounting for 17%.

The proportion of malignant tumours from all uveal tissue increased from 11% (including one choroidal fibrosarcoma and one iris melanoma) and 17% (two iris melanomas) in the first two intervals to 28% (comprising ten iris melanomas and seven ciliary body melanomas) and 33% (consisting of eight iris melanomas, two ciliary body melanomas, and four choroidal melanomas) in the last two intervals.

While only one case of *iris melanoma* was recorded in the interval from 1945 to 1954, it has since become one of the two most common uveal diagnoses in each subsequent interval, accounting for 9.5% (*n* = 2) in 1985–1994 and 19% (*n* = 8) in 2005–2015. The median age of patients at the time of surgery varied between 36 years (1985–1994) and 72 years (2005–2015) (Table 2).

*Normal iris* has consistently been among the “Top 5” diagnoses in each interval. Initially, there was only one case in each of the first two intervals (5.6% in 1945–1954 and 8.3% in 1955–1964). However, the absolute numbers increased over time, reaching 18 diagnoses in 1995–2004, which also marked its highest relative percentage of 30%. The median age of patients at the time of surgery ranged from 10 years (1955–1964) to 70 years (1985–1994) (Table 2).

### Intraocular tissue unspecified

A total of 198 intraocular tissue samples and 60 evisceration specimens were received for histopathological examination; however, the diagnoses of the latter were not analyzed further in this study. The primary reason for the removal of intraocular tissue was *trauma*, with the exception of three cases: one case of bacterial endophthalmitis (1995–2004), one case involving fibrin, and one case of purulent endophthalmitis (both occurring in 2005–2015) (Table 1). No intraocular components were excised during the first two intervals, and only three specimens were removed in the subsequent 20 years (1965–1984). However, this was followed by a significant increase in the number of cases: 43 in the period 1985–1994, 82 in 1995–2004, and 70 in 2005–2015. Given the prognostic significance of this differentiation, we distinguished between trauma-associated tissue with and without retinal components. The ratio of trauma-associated tissue without retinal components to those with retinal components increased from 2:1 in the period 1985–1994 to 3:1 in 1995–2004, and further to 4:1 in 2005–2015 (Table 1). The median age at surgery increased from 17.5 years (1965–1974) to 63.5 years for trauma-associated tissue without retinal components, and to 72 years for such tissue with retinal components, with both observations noted during the period 2005–2015 (Table 2).

### Vitreous

178 vitreous samples were analysed, with the primary diagnosis overall being vitritis (47%), followed by normal vitreous (15%). While no cases were recorded in the first interval, only one case was noted in the second interval and two in the third. These numbers subsequently increased to 25 cases in the period 1985–1994 and further rose to over 70 cases in each of the last two intervals (Table 1).

Since its first archived diagnosis in the period 1975–1984, *vitritis* has consistently remained the most common vitreous diagnosis. Its prevalence has ranged from 33% (*n* = 2) in 1975–1984 to 52% (*n* = 13) in 1985–1994 (Table 1). The median age of patients at the time of surgery varied between 32.5 years (1975–1984) and 73 years (2005–2015) (Table 2).

At the beginning of the observational period, *normal vitreous* was the most common vitreous diagnosis, with one case recorded in both 1955–1964 and 1965–1974. Subsequently, it remained the second most common diagnosis, with its prevalence ranging from 12% (*n* = 9) in 2005–2015 to 17% (*n* = 1) in 1975–1984 (Table 1). The median age of patients at the time of surgery varied between 16 years (1975–1984) and 70 years (2005–2015) (Table 2).

A diagnosis of *lymphoma* in vitreous samples was first documented in our archive in 1993. Lymphoma accounted for 5.5% (*n* = 4) of all vitreous diagnoses during the period 1995–2004, and 8.5% (*n* = 6) in 2005–2015. Additionally, during the latter period, lymphoma could not be excluded in seven cases.

### Lacrimal gland

Throughout our study period, 144 lacrimal gland samples were submitted for histological analysis. The most frequent diagnoses were dacryoadenitis (28%), followed by normal lacrimal gland (16%). Malignant tumours accounted for 15% of all lesions (*n* = 22), including eight adenoid cystic carcinomas, seven lymphomas, two malignant mixed tumours, two poorly differentiated carcinomas, one undefined carcinoma, one melanoma, and one undefined malignant tumour. Among these, epithelial neoplasms were the most prevalent, comprising 59% of the cases. The number of lacrimal gland samples increased almost steadily, from two cases of hypertrophy in the period 1945–1954 to 45 diagnoses in the final interval, 2005–2015 (Table 1).

Since the period 1965–1974, *dacryoadenitis* has consistently ranked among the three most common diagnoses. Its prevalence has ranged from 8.3% (*n* = 1) in 1965–1974 to 57% (*n* = 21) in 1995–2004. *Normal lacrimal gland* was the most prevalent diagnosis in the periods 1955–1964 (50%, *n* = 2) and 2005–2015 (29%, *n* = 13). The median age of patients at the time of surgery ranged from 42 years (1985–1994) to 55 years (1965–1974) for dacryoadenitis, and from 17.5 years (1955–1964) to 73 years (1975–1984) for normal lacrimal gland (Table 2).

### Retina

Over the course of our study, a diagnosis of retinal tissue was documented in 93 cases. The most prevalent diagnoses were subretinal choroidal neovascularization (CNV, 23%), followed by epiretinal gliosis (17%), all of which were identified after 1989. Initially, no specimens were submitted for histological examination at the beginning of the observational period, and only four cases were recorded between 1975 and 1984. However, the number of cases increased significantly to 34 and 46 during the last two intervals, which accounted for 86% of all retinal tissue samples submitted. In the period 1985–1994, the most common retinal diagnosis was *proliferative vitreoretinopathy* (PVR, 33%, *n* = 3), followed by bleeding (22%, *n* = 2). During 1995–2004, *CNV* (41%, *n* = 14) was the leading diagnosis, followed by *epiretinal gliosis* (18%, *n* = 6) and *PVR* (12%, *n* = 4). In the final interval, 2005–2015, epiretinal gliosis (20%, *n* = 5) was the most frequent diagnosis, followed by CNV (15%, *n* = 7) (Table 1). Patients diagnosed with CNV had median ages ranging from 74 years during the period 1995–2004 to 81 years in 2005–2015. Conversely, for those with PVR, the median age ranged from 51.5 years in 2005–2015 to 75 years in 1995–2004 (Table 2).

### Exenteration

Over the observed time span of 71 years, a total of 59 exenterations were performed, with malignant tumours identified in 81% of all specimens. BCC was the most prevalent diagnosis, accounting for 17% of cases, followed by SCC at 10%. The number of diagnoses per interval did not exhibit a noticeable increase throughout the observation period but remained relatively constant, ranging from four cases in 1955–1964 to 15 cases in 1985–1994 (Table 1). In the two instances where normal findings were reported, the reasons for performing exenteration were SCC of the maxilla and blindness due to sphenoid wing meningioma, both occurring in 1981. Out of the three exenteration specimens with resection after R1, one patient was found to be tumour-free following the resection of a malignant eyelid melanoma in 2003. Similarly, another patient was tumour-free after the resection of a Meibomian gland carcinoma in 2007, and a third patient was tumour-free following the resection of a conjunctival melanoma in 2008.

The first case of *BCC* was recorded in the period 1945–1954, followed by another case in 1975–1984. The incidence increased to seven diagnoses (47%) in 1985–1994 (Table 1), making it the most common diagnosis during that interval. However, BCC did not feature among the most frequent diagnoses in 1995–2004, and only one case (13%) was identified in 2005–2015. With just three specimens, *SCC* was among the three most common diagnoses in the years 1945–1954 (11%, *n* = 1) and 1955–1964 (50%, *n* = 2). It was the second most frequent diagnosis with seven cases in 1985–1994 (47%). The age of patients at the time of exenteration ranged from 44 years (1945–1954) to 93 years (2005–2015) for BCC, and from 48 years (1945–1954) to 84 years (2005–2015) for SCC (Table 2).

### Sclera

Among all the registered 38 scleral samples, the most frequent diagnosis was normal sclera (32%), which was removed during trabeculectomy, followed by scleritis (27%).

The diagnosis of *normal sclera* was first recorded in the period 1965–1974 (*n* = 1), with the number of cases not exceeding six per interval (1995–2004) throughout our study period (Table 1). Despite this, normal sclera was the most frequent diagnosis in the intervals 1975–1984 (50%, *n* = 2) and 1995–2004 (60%, *n* = 6). Additionally, *scleritis* was among the three most common diagnoses in the years 1955–1964 and from 1975 to 1984 to 2005–2015, although the number of cases did not exceed four per interval (2005–2015). The age of patients at the time of diagnosis ranged from 42 to 81 years for normal sclera and from 10 to 77 years for scleritis (Table 2).

### Anterior chamber angle

During our study period, a total of 37 specimens from the anterior chamber angle were submitted for analysis. The most prevalent diagnosis was sclera and trabecular meshwork, which were removed during filtering glaucoma surgery, accounting for 59% of the cases. This was followed by inflammation, which constituted 8.1% of the diagnoses. The first instances of sclera and trabecular meshwork specimens were documented in the period 1965–1974 (*n* = 3, 100%) (Table 1). This was followed by an additional 14 specimens (67%) in 1975–1984 and five such specimens (71%) in the same interval, 1975–1984. 

Within these intervals, *sclera* and *trabecular meshwork* were also the most frequently submitted specimens from the anterior chamber angle. No specimens from this topographical area were examined in the period 1995–2004. However, six specimens were submitted for histological examination in 2005–2015, of which two (33%) were diagnosed with inflammation of the trabecular meshwork (Table 1). Other diagnoses included normal and pigmented trabecular meshwork, trabecular meshwork with vacuoles, and connective tissue (each *n* = 1, 17%). The age of patients at the time of anterior chamber angle surgery ranged from 9 to 82 years. For sclera and trabecular meshwork, the age range differences increased significantly, from 12 years (62–74) in 1965–1974 to 67 years (15–82) in 1985–1994 (Table 2).

### Anterior chamber

A total of 27 samples from the anterior chamber have been archived in our database. The most common diagnoses were epithelial downgrowth (52%) and inflammation (26%). Epithelial downgrowth was the dominant diagnosis until the period 1975–1984 (*n* = 7, 100%), whereas inflammation of the anterior chamber emerged as the most frequent diagnosis during the last two intervals, with three specimens recorded in each (Table 1). Notably, half of the epithelial downgrowth samples were characterized as cystic. The age range of patients undergoing anterior chamber surgery for epithelial downgrowth spanned from 28 to 81 years. In contrast, inflammation was observed in patients aged between 44 and 84 years (Table 2).

### Optic nerve

Specimens from the optic nerve were the least commonly submitted, with only 15 cases undergoing further histological analysis over the entire 71-year study period. The most frequent diagnoses were meningioma, glioma, atrophic optic nerve, and normal optic nerve, each accounting for three cases (20%). Benign and malignant neoplasms constituted 53% of all optic nerve specimens. No samples were recorded in our database for the periods 1945–1954, 1985–1994, and 2005–2015. The first documented case of an optic nerve specimen diagnosed with atrophy was recorded between 1955 and 1964 (Table 1). In the interval 1965–1974, two cases of glioma (40%), one case of atrophy (20%), and one normal optic nerve specimen (20%) were submitted. During 1975–1984, four optic nerve specimens were examined, of which two were normal, one was diagnosed as meningioma, and another as glioma. Between 1995 and 2004, histological examination was performed on five optic nerve samples, including two meningiomas, two malignant nerve sheath tumours, and one case of atrophy. The age of patients at the time of surgery ranged from 15 to 61 years for those diagnosed with meningioma, 5 to 29 years for glioma, and optic nerve atrophy was observed in patients aged between 5 and 62 years (Table 2).

## Discussion

We have conducted an extensive retrospective analysis of all archived ocular pathology reports from the Specialised Ophthalmic Pathology Laboratory at the Eye Center, Medical Center, University of Freiburg, covering a period of over seven decades. Recently, we published the results of the *localisation* of all examined specimens and *categories* from main topographical areas [20]. For this study, we examined the changes in histological *diagnoses* from all locations, including the less frequent topographical regions, to provide a more detailed insight into the trends and patterns of ocular diseases. Only the study by Rohrbach et al. has examined the shift in the type and frequency of histological diagnoses over time [8]. However, they did not cover a continuous time period, but instead focused on single years with 20- and 10-year intervals between 1900 and 1990, analysing these changes confined to the localisations of the eyelid and cornea.

Between 1945 and 2015, the period under observation saw a steady rise in patient numbers, driven by population growth and an expanding catchment area (Figs. 1–4). Concurrently, an increasing number of ophthalmic surgeons collected more patient samples for histological examination. This general upward trend was further shaped by historical events, advancements in medical techniques, and changes in leadership.

Initially, the expansion of laboratory capacity, particularly in terms of the number of specimens received for histological examination, was hampered by the aftermath of the Second World War, particularly the destruction of the clinic in November 1944. However, the opening of the newly constructed Freiburg Eye Center in 1964 and the introduction of microsurgical techniques in the late 1960s by Mackensen (director from 1968 to 1987) marked a significant turning point (Figs. 1–3). These developments facilitated more frequent excisions, particularly from ocular sites. The number of samples submitted for histological examination continued to surge under Witschel’s leadership (1988–2002). This trend continued under the directorship of Reinhard, which began in 2003. During this period, annual submissions not only far surpassed previous levels but also placed a notable emphasis on corneal and conjunctival specimens (Figs. 2 and 3). Additionally, since the mid-1990s, an increase in external submissions from other institutions has contributed to the growing sample numbers, reflecting the clinic’s broadening regional influence and supporting a steady rise from 1998 onward. Another notable observation is the widening age range of patients over the study period (Table 2). While this could be a statistical effect due to the inclusion of more patients, it may also result from earlier interventions made possible by improved surgical techniques and an aging population.

### Eyelid

The eyelid was the most common site for histopathological diagnoses. Initially, the three primary eyelid diagnoses were dominated by malignant tumours, specifically squamous cell carcinoma (SCC) and basal cell carcinoma (BCC). Over time, however, a noticeable shift occurred towards benign conditions such as papillomas and chalazia, with the latter becoming the leading diagnoses in the final two intervals from 1995 to 2015 (Table 1). This trend may be attributed to the prioritisation of clinically suspected malignant lesions in the aftermath of the Second World War, a period during which surgeries and histological examinations were limited. Additionally, the increasing emphasis on cosmetic reasons for the removal of eyelid lesions may explain the rise in the examination of benign tumours, particularly papillomas and chalazia (Fig. 1). Another contributing factor to the increasing numbers of chalazia is that, since Heinrich Witschel’s directorship, all such cases have been sent for histological examination. However, according to personal communications with members of the European Ophthalmic Pathology Society (EOPS), this practice is not standard in other countries.

Throughout our entire observational period, *chalazion* emerged as the most frequent eyelid diagnosis, constituting 18% of all cases. Two distinct age ranges for chalazion removal are particularly noticeable (Fig. 5). The first range spans from two to seven years, peaking at age 4. This period coincides with the kindergarten years and the beginning of school. We assume that interventions during this period were primarily aimed at preventing children from being teased by their peers. The second range begins beyond age 12, with 10 cases, increasing to 63 cases by age 17, where it reaches a plateau that extends until age 40. This rise is presumably due to an increasing awareness of the cosmetic effects associated with the condition. For the next four most frequent diagnoses depicted in Fig. 5, the expected gradual increase is observed – steeper in the case of BCC – until, after reaching their peak between patient ages of 59 and 80, the age distribution curves descend more or less sharply.

While *BCC*, with an overall proportion of 16%, consistently ranked among the top three most frequent diagnoses throughout the study, its proportion decreased from 26% in the third interval to 15% by the final observation period. This decline can be primarily attributed to an increasing number of chalazia diagnoses (Table 1). Furthermore, the relative frequency of SCC experienced a dramatic decline from 22% in the initial interval to 12% during the period from 1955 to 1964, and further to just 1.1% in the third interval (1965–1974). We speculate that UV protection, particularly through the use of sunscreen, may be more effective in preventing SCC than BCC. At the same time, younger patients have increasingly been exposed to UV radiation without adequate protective measures. This could explain the observed decrease in the frequency of SCC, while concurrently, the youngest age at SCC surgery more than halved from 45 years in 1945–1954 to 21 years in 1995–2004. Similarly, throughout our study period, the age range of patients undergoing surgical removal of BCC expanded significantly, from 32 to 80 years. The youngest patient was 46 years old in the period from 1945 to 1954, dropping to just 18 years old in the interval from 1975 to 1984 (Table 2). Both observations regarding SCC and BCC can be attributed to increased life expectancy and potentially prolonged exposure to UV radiation, impacting a growing number of younger patients. These individuals may have experienced increased sun exposure during their childhood and adolescence, typically without consistent sun protection.

We attribute the observed decline in the relative frequency of *papillomas*, from 26% in the period 1975–1984 to 11% in the final interval (1995–2015) (Table 1), to the concurrent rise in the number of chalazia cases, which increased from 166 to 2,293. However, the overall percentage of papilloma diagnoses is equal to that of chalazia, both constituting 16%. While the range of patient ages undergoing papilloma surgery expanded dramatically from 38 years in the initial observation interval to 98 years in the 1965–1974 interval, the age of the youngest patient decreased from 37 years to just 2 years over the same period (Table 2). This shift can be attributed to growing cosmetic awareness within the population and advancements in surgical care, particularly since papilloma surgery is conducted under general anaesthesia for patients under the age of 5.

The overall percentage of resection after R1 was 6.5% across our study. One possible explanation for the observed significant increase in further resections, primarily following *incomplete surgical removal of BCC* since 1991, is a shift towards more tissue-conserving surgeries. This increase is notable, with rates rising from 1.3% (*n* = 39) in the 1985–1994 interval to 10% (*n* = 1,025) in the most recent interval, despite a concurrent decrease in the frequency of BCC (Table 1). Instances of tumour-free resections may be attributed to changes in the types of BCC that more frequently necessitate additional resections. These subsequent resections often do not contain residual tumour tissue, potentially due to its destruction by postoperative inflammation [21].

Although the absolute number of *nevus* diagnoses, which constituted an overall proportion of 4.6% of all eyelid cases, increased from two cases to 349 over our observation period (Table 1), their decreasing proportion may be explained by the concurrent rise in the number of chalazia diagnoses. This trend mirrors that observed with papillomas. Nevi formation is, among other factors, influenced by genetic predisposition and skin type. Fair skin, with less protective melanin, heightens the risk of developing these benign pigmented lesions. In our study, the age range of patients has steadily increased over the observed periods. From 1945 to 1954, it spanned 35 years, with the youngest patient undergoing surgery at 14 years of age and the oldest at 49 years. By our most recent interval, this range had expanded to 89 years, with the youngest patient being 5 years old and the oldest 94 years old (Table 2). This widening age range may indicate increased willingness to undergo surgery regardless of age, which is depicted by the broad and flat age distribution curve for nevi in Fig. 5.

Dermoid cysts, which are congenital lesions, represented 1.1% of all eyelid surgeries. Throughout the study period, their contribution to eyelid lesions peaked at 4.8% between 1965 and 1974. Subsequently, however, their relative frequency decreased to below 1.4% in our most recent interval (Table 1). Over the course of the study, there was a notable shift in the median age at the time of surgery. Initially, the median age was among adults (37 years; 1945–1954), but it shifted to adolescents (17 years; 1955–1964) and further decreased to preschool-aged children (from 6 to 4 years; 1975–2015) (Table 2). This shift can largely be attributed to the efforts of parents to prevent school-aged children from experiencing peer teasing have contributed to this trend.

We will now compare our findings for the three most common diagnoses in eyelid specimens – chalazia, BCC, and papilloma – with those reported in the available literature. This comparison will utilize the relative frequencies of these diagnoses in Freiburg, within time intervals adjusted to align with those of the comparative studies (Table 3).

**Table 3:** Comparison of eyelid diagnoses with results from other studies

Study	Region	Time period	Chalazion (%)	BCC (%)	Papilloma (%)
Rohrbach et al. [8]	Tübingen (DE)	1940	3	0	-
*Our study*	Freiburg (DE)	1945	0	0	
Rohrbach et al. [8]	Tübingen (DE)	1960	9	22	-
*Our study*	Freiburg (DE)	1960	0	19	
Rohrbach et al. [8]	Tübingen (DE)	1980	34	14	-
*Our study*	Freiburg (DE)	1980	9	20	
Rohrbach et al. [8]	Tübingen (DE)	1990	34	7	-
*Our study*	Freiburg (DE)	1990	16	19	
Deprez and Uffer [15]	Lausanne (CH)	1989–2007	38	14^a^	-
*Our study*	Freiburg (DE)	1989–2007	22	17	
Aurora and Blodi [9]	Iowa City (US)	1932–1969	12	19	18^b^
*Our study*	Freiburg (DE)	1945–1969	6.9	21	17
Spraul and Grossniklaus [10]	Atlanta (US)	1941–1995	16	10	21
*Our study*	Freiburg (DE)	1945–1995	11	20	23
Welch and Duke [11]	Baltimore (US)	1952–1956	13	11	25
*Our study*	Freiburg (DE)	1952–1956	10	11	27
Wang et al. [13]	South China	2000–2018	1.9	7.3	21
*Our study*	Freiburg (DE)	2000–2015	25	17	14
Domingo et al. [14]	Philippines	2003–2012	1.9	9.8	15
*Our study*	Freiburg (DE)	2003–2012	26	16	13

This table presents a comparison of the relative frequencies of the three most prevalent eyelid diagnoses in our study with those reported in existing literature

^a^ Inflammatory lesions are not included

^b^ Seborrheic keratosis

Rohrbach et al. [8] reviewed the histological diagnoses on 1,835 specimens collected in 20- or 10-year increments between 1900 and 1990 at the ophthalmic pathology laboratory at the University Eye Hospital Tübingen, Germany. The authors reported an increase in relative frequency of *chalazia*: 3% in 1940, 9% in 1960, 34% in 1980, and 34% in 1990 (Table 3). In Freiburg, the annual data reveal figures of 0% in 1945, 0% in 1960, 9% in 1980, and 16% in 1990. Similarly, our interval-based results also indicate an increase, albeit less pronounced than in Tübingen, with percentages of 5.5% in 1955–1964, 7.5% in 1975–1984, and 14% in 1985–1994. This upward trend continues, reaching 20% in 1995–2004 and 22% in 2005–2015 (Table 1), peaking at 29% in 2004. This peak is similar to the findings reported by Rohrbach et al. in 1980 and 1990.

Deprez and Uffer [15] evaluated 5,504 eyelid tumours collected between 1989 and 2007 in the Laboratory of Ophthalmopathology of the Hôpital Ophtalmique Jules Gonin, Lausanne, Switzerland. Chalazia occurred with a relative frequency of 38%, nearly matching the findings of Rohrbach et al. [8] and significantly exceeding our own results of 22% within the 1989–2007 period (Table 3).

In their study, Aurora and Blodi [9] evaluated 892 eyelid tumours collected from 1932 to 1969 at the Department of Ophthalmology, University of Iowa, College of Medicine, Iowa City, IA, USA. They reported a proportion of 12% chalazia, whereas we found 6.9% within the same time interval (Table 3). Two additional studies from USA report relative frequencies for chalazion that are closer to our own findings. Spraul and Grossniklaus [10] analysed 24,444 surgical ocular and periocular specimens from the L.F. Montgomery Ophthalmic Pathologic Laboratory at Emory University, Atlanta, GA, USA, collected from 1941 to 1995. They reported a frequency of 16%, compared to our own 11% within the same time frame (Table 3). Welch and Duke [11] reviewed 617 eyelid tumours collected from 1952 to 1956, many of which were examined at the ophthalmic pathology laboratory at the Wilmer Ophthalmological Institute of The Johns Hopkins University and Hospital, Baltimore, MD, USA. Both authors found a frequency of 13, compared to our 10% within the same study interval.

In two Asian studies, the proportion of chalazion was considerably lower. The first study is that of Wang et al. [13], who analysed 5,146 eyelid tumours and tumour-like lesions collected from 2000 to 2018 at the Ophthalmology Department of the Second Affiliated Hospital, Zhejiang University School of Medicine (ZJU-2), South China. Domingo et al. [14] reviewed 1,551 tumours comprising 530 from the eyelids, 254 from the conjunctiva, 394 from intraocular locations and 373 from the orbit at the Ocular Pathology Section of the Philippine Eye Research Institute, Manila, Philippines, archived in the period 2003 to 2012. Both studies reported a relative frequency of only 1.9% for chalazion among the South Chinese and the Philippine patients who underwent chalazion removal surgery, followed by histopathological diagnosis, compared to our findings of 25% (2000–2015) and 26% (2003–2012) (Table 3). It remains unclear whether all excised chalazia were examined histologically or only those deemed suspicious. In contrast, our data includes all chalazion specimens excised at our institution. Furthermore, the low incidence in the Philippine study population might be explained by differences in socioeconomic factors and health insurance coverage, which influence patients’ lifestyles and their willingness to undergo surgery for cosmetic reasons. Furthermore, there may be genuine differences in the incidence of Meibomian gland dysfunction between Caucasian patients and those of Asian descent, including Chinese and Filipino individuals.

Consistent with our 19% proportion of *BCC* cases in 1960, Rohrbach et al. [8] reported 22% in Tübingen for the same year. This was followed by a decrease to 14% in 1980 and 7% in 1990, which contrasts with our findings of 20% in 1980 and 19% in 1990 in Freiburg (Table 3). This discrepancy may be attributed to differences in the availability of plastic surgeons, as reconstruction following BCC removal can be technically challenging compared to chalazion surgery.

Aurora and Blodi [9], Spraul and Grossniklaus [10], and Welch and Duke [11] conducted separate studies involving three distinct populations from the United States. They reported relative frequencies of BCC cases at 19% in Iowa City (1932–1969), 10% in Atlanta (1941–1995), and 11% in Baltimore (1952–1956). These figures are comparable to our findings in Freiburg of 21% (1945–1969), 20% (1945–1995), and 11% (1952–1956). Differences between the frequencies reported from Atlanta [10] and Freiburg might be attributed to variations in surgical capacities and priorities among the different centres.

BCC accounted for 7.3% of all eyelid lesions in a large Asian series from South China, involving specimens excised between 2000 and 2018 [13], and 9.8% in a Philippine study population from 2003 to 2012 [14]. In comparison, our study observed frequencies of 17% (2000–2015) and 16% (2003–2012) (Table 3). Asians exhibit a higher susceptibility to other malignant eyelid tumours, particularly sebaceous gland carcinoma. This increased susceptibility, along with a higher natural UV resistance due to increased melanin production, might explain the lower proportion of BCC [13]. The presence of a certain proportion of patients of Asian origin within their study population could provide an additional explanation for the lower frequency of BCC reported in the study from Atlanta [10].

On the other hand, the population composition in Lausanne is likely more similar to that in Freiburg. Deprez and Uffer [15] reported a relative frequency of 14% between 1989 and 2007, compared to our 17% for the same period. However, their study focused solely on eyelid tumours and excluded inflammatory lesions.

In this study, we did not stratify cases by geographic location. However, based on our comparative analysis with data from Tübingen [8]—a tertiary eye care centre located 110 km northeast of Freiburg—we observed moderate diagnostic variations in chalazion and BCC (Table 3) and in keratoconus (Table 4), depending on the observed time period. Chalazion and BCC showed a more limited variation between Freiburg and Lausanne [15] (a tertiary eye care centre located 190 km southwest of Freiburg), while the Lausanne findings of chalazion almost matched those in Tübingen (Table 3). Although Freiburg is located approximately 1,586 km further from the equator than Atlanta (USA), similar variations can be found in all three above mentioned diagnoses [10] (Tables 3 and 4), as we detected between Tübingen, Freiburg and Lausanne.

**Table 4:** Comparison of corneal diagnoses with results from other studies

Study	Region	Time period	Fuchs’ dystrophy (%)	Keratoconus (%)	Keratitis (%)	Transplant failure (%)
Rohrbach et al. [8]	Tübingen (DE)	1940		0		
*Our study*	Freiburg (DE)	1945		0		
Rohrbach et al. [8]	Tübingen (DE)	1960		8		
*Our study*	Freiburg (DE)	1960		17		
Rohrbach et al. [8]	Tübingen (DE)	1980		27		
*Our study*	Freiburg (DE)	1980		30		
Rohrbach et al. [8]	Tübingen (DE)	1990		28		
*Our study*	Freiburg (DE)	1990		20		
Rahman et al. [22]	Manchester (UK)	2007–2008	15.4	21.7	5.8^a^	22.3
*Our study*	Freiburg (DE)	2007–2008	19	19	12	17
Spraul and Grossniklaus [10]	Atlanta (US)	1941–1995	16^b^	11	19	
*Our study*	Freiburg (DE)	1945–1995	8	19	14	
Gosheh et al. [23]	Philadelphia (US)	2001–2005	10.8	16	2.7^c^	22.0
*Our study*	Freiburg (DE)	2001–2005	19	18	17	10
Kelly et al. [24]	Australia	1985–2009		31		19
*Our study*	Freiburg (DE)	1985–2009		19		11
Keane et al. [25]	Australia	1985–2020	18	24	3.7^d^	24
*Our study*	Freiburg (DE)	1985–2015	24	15	12	13
Edwards et al. [26]	New Zealand	1991–1999	4.4	45.6	7.3^e^	8.7
*Our study*	Freiburg (DE)	1991–1999	9.2	22	12	7.1
Cunningham et al. [27]	New Zealand	2000–2009	8.2	41.1	7.9	17.0
*Our study*	Freiburg (DE)	2000–2009	19	18	15	13
Xie et al. [28]	North China	1997–2002		12.9	31.8	4.5
*Our study*	Freiburg (DE)	1997–2002		23	15	4.5
Matthaei et al. [29]	Europe	1980–2014	10.2	24.2	13.2	
	North America	1980–2014	12.9	14.2		16.3
	Asia	1980–2014		8.1	32.5	11.1
*Our study*	Freiburg (DE)	1980–2014	20	16	13	13

This table presents a comparison of the relative frequencies of the four most prevalent corneal diagnoses in our study with those reported in existing literature

^a^ Ulcerative and infectious keratitis

^b^ 92.6% of all corneal dystrophies which represented 16.8% of all cornea buttons excised during penetrating keratoplasty

^c^ Viral keratitis

^d^ Clinical diagnosis, histologically not confirmed

^e^ Herpes simplex and herpes zoster keratitis

The relative frequency of *papilloma* cases is reported in five of the studies listed in Table 3: 18% in Iowa City (1932–1969) [9], 21% in Atlanta (1941–1995) [10], and 25% in Baltimore (1952–1956) [11]. These figures closely align with our findings in Freiburg of 17% (1945–1969), 23% (1945–1995), and 27% (1952–1956). Wang et al. [13] reported a similar proportion of 21% for papilloma among eyelid tumours and tumour-like lesions in their South Chinese patient cohort during the more recent time interval of 2000–2018. In contrast, our findings in Freiburg indicate a lower proportion of 14% within the period of 2000–2015. However, the proportion of 15% reported by Domingo et al. [14] between 2003 and 2012 aligns closely with the relative frequency of papilloma cases (13%) histologically diagnosed in Freiburg during the same period (2003–2012).

### Cornea

Corneal tissue was the second most frequent topography, with *Fuchs’ dystrophy* being the most common associated diagnosis, accounting for 19% of cases. Since its initial appearance in the “Top 10” corneal diagnoses with an 8% proportion during the 1965–1974 interval, the frequency of Fuchs’ dystrophy has steadily increased to 12% in the period from 1995 to 2004 (Table 1). This rise can be attributed to Mackensen, who, after assuming directorship in Freiburg in 1967, introduced ocular surgery under the microscope. Improvements in surgical techniques also come into play here. Since 2003, under the directorship of Reinhard, a specialist in corneal diseases, and with the support of our Lions Eye Bank in Freiburg, there has been a significant increase in the numbers. Specifically, the figures have risen to 1,082, representing a 25% proportion during the interval from 2005 to 2015, and 79% of all cases of Fuchs’ dystrophy within our study period. Furthermore, the Descemet’s membrane endothelial keratoplasty (DMEK) procedure, introduced by Melles in 2008 [30], offers a faster surgical process and visual rehabilitation compared to perforating keratoplasty (PKP). In our ophthalmic pathology laboratory, we observed a substantial increase in Descemet’s membranes, from 23 specimens in 2011 to 299 in 2015. This surge contributed to a significant increase in specimens diagnosed with Fuchs’ dystrophy, which rose from 56 to 262 over the same five-year period (Fig. 2). These numbers account for 57% of all Fuchs’ dystrophy specimens diagnosed during our entire study interval. In summary, it can be stated that the introduction of the DMEK procedure, combined with enhanced clinical expertise and resources, has allowed for earlier establishment of surgical indications. This advancement has led to an increase in the number of Descemet’s membrane cases, particularly those diagnosed with Fuchs’ dystrophy, being submitted in Freiburg.

In our study, *keratoconus* was the second most common corneal diagnosis, accounting for 13% of cases. The first two specimens were excised in 1960 and 1961, each representing 17% of the diagnoses. Following this, there was an increase in the relative frequency, reaching a peak of 30% in 1980. The slight decrease in the proportion of keratoconus following the 1975–1984 interval (Table 1) can most likely be attributed to two key advances in treatment. Firstly, improvements in the fitting of dimensionally rigid contact lenses delayed the need for penetrating keratoplasty, and secondly, the introduction of corneal collagen cross-linking in 1997 further contributed to this trend [31, 32]. Following the introduction of DMEK in 2007, the substantial increase in cases of Fuchs’ dystrophy led to a decline in the proportion of keratoconus cases in Freiburg. However, the actual number of keratoconus cases continued to rise, reaching 446 in the final study interval. The continuous increase in the median age of patients, which doubled from 21 years (1955–1964) to 41 years (2005–2015) over the majority of our observation period (Table 2), can be attributed to advancements in rigid contact lens fitting and cross-linking procedure. These improvements have resulted in extended time spans before surgery becomes necessary. Unlike Fuchs’ dystrophy, keratitis, transplant failure, and scar tissue – conditions where the peak age for surgery ranges between 65 and 78 years – keratoconus predominantly affects younger individuals. Consequently, the peak age for keratoconus surgery is 28 years (Fig. 6). The progression of keratoconus varies; some cases deteriorate rapidly, necessitating earlier surgical intervention, while others follow a milder course, delaying the need for surgery until later in life.

Our study identified *keratitis* as the third most prevalent corneal diagnosis, comprising 11% of all cases. During the first four intervals, we observed a higher relative frequency of keratitis (22%, 27%, 11%, 19%) requiring keratoplasty prior to 1986 (Table 1). This was followed by a decline to a range of 9.4% to 14% during the last three intervals. Several factors could account for this trend: improvements in antibiotic medications, changes in the treatment of bacterial keratitis – such as the adoption of thermocauterisation [33], introduced in Freiburg by Reinhard in 2003 – and an increase in the diagnosis of other conditions such as Fuchs’ dystrophy. Whereas, although not ranked in the “Top 5”, the proportion of corneal infections – defined as specimens with histologically confirmed microbes such as fungi and Acanthamoeba, or clinically unequivocal herpetic infections – has remained relatively consistent. After a peak of 8.2% in the period from 1955 to 1964, the subsequent intervals show a proportion of 1.2–4.3% corneal infections.

At the beginning of our observation period (1945–1954), the youngest patient with corneal inflammation was 24 years old (Table 2). The youngest age decreased to less than 6 months, recorded as 0 years, during the periods from 1975 to 1984 and 1985 to 1994. This occurred in three instances: once in 1976, once in 1980, and again in 1986. Similarly, the youngest patient with a histologically confirmed corneal infection was 41 years old during the interval from 1955 to 1964. By the period from 2005 to 2015, this youngest age had also dropped to 1 year. Both can be attributed to the enhanced expertise in corneal surgery in Freiburg since 2003.

*Transplant failure*, similar to keratitis, accounted for 11% of all corneal histological diagnoses. During the initial four intervals leading up to 1984, their relative frequency varied between 10 and 15% (Table 1). An exception to this trend was noted between 1955 and 1964, where a lower incidence of 6.1% was observed, likely due to a higher proportion of keratitis (27%) and infections (8.2%) during that period. The subsequent decrease in percentages during the intervals from 1985 to 1994 (5.9%) and 1995 to 2004 (5.3%) can be attributed to the implementation of more intensive post-operative anti-inflammatory therapies. These include consistent long-term use of low-dose topical steroids and systemic immunosuppression in high-risk keratoplasties. In the final interval of our study, during which 75% of all transplant failure diagnoses were made, we noted an increased relative frequency of 14% (Table 1). This rise could potentially be explained by the overall increase in the number of keratoplasties, leading to more frequent graft failure. The introduction of DMEK has enabled earlier indications for re-keratoplasty in cases of transplant failure due to endothelial loss. However, during the most recent years analysed, we have not observed an increase in failed transplants; instead, there has been a decrease from 20% in 2011 to 12% in 2015. This trend may be associated with advancements in DMEK techniques, which have resulted in a reduced frequency of graft rejection and subsequent transplant failure.

Initially, during the period from 1945 to 1954, the youngest patient diagnosed transplant failure was 23 years old (Table 2). By the last interval of our study, from 2005 to 2015, the age of the youngest patient had decreased significantly to just 3 years. Similar to keratitis surgery, this significant change can likely be attributed to advancements in corneal surgical techniques.

*Scar tissue* removal constituted 10% of all corneal cases. Over the course of our observation period, its relative frequency typically ranged from 7.2% to 16%, with a notable peak of 29% occurring between 1965 and 1974 (Table 1). The subsequent significant decline to 7.2% during 1995–2004 can be attributed to several factors. The introduction of mandatory seat belt laws in Germany on January 1, 1976, likely contributed significantly to the decrease in the relative frequency of accident-related corneal scars, which fell from 29% to 16% between 1975 and 1984. Enhanced safety measures, in accordance with the Occupational Health and Safety Act enacted in Germany in 1996, including the mandatory use of safety goggles, may have further reduced the percentage from 12% in 1985–1994 to 7.2% in 1995–2004. Additionally, throughout our study period, advancements in therapy options for keratitis have likely contributed to the overall reduction in the occurrence of corneal scars.

As previously noted with corneal infections and inflammations, the median patient age at surgical removal of scar tissue increased from 33 years (1955–1964) to 61 years (2005–2015) (Table 2). Concurrently, the youngest patient’s age decreased from 18 years (1955–1964) to just 1 year (2005–2015). These changes can be attributed to the advanced expertise in corneal surgery available in Freiburg, enabling treatments to be conducted regardless of the patient’s age.

A comparative overview of our results and those from existing literature is presented in Table 4. Notably, a proportion of 79% of *Fuchs’ dystrophy cases* were histologically diagnosed in our Specialised Ophthalmic Pathology Laboratory during the most recent study interval (2005–2015). The substantial number of cases occurring in the most recent period may account for some of the discrepancies observed when compared to other studies that cover periods prior to 2005. Rahman et al. [22] reviewed 203 cases of penetrating keratoplasties conducted between 2000 and 2003 at Manchester Royal Eye Hospital, Manchester, UK. Their observed proportion of Fuchs’ dystrophy, at 15.4%, is comparable to our findings of 19% within the same time interval (Table 4). In contrast, Spraul and Grossniklaus [10] reported a relative frequency in Atlanta in 1941–1995 which was twice as high as ours, at 16% compared to 8% in Freiburg in 1945–1995. The higher proportion observed in Atlanta is likely attributable to the inclusion of excised corneas and corneas from enucleated bulbi. Gosheh et al. [23] carried out a retrospective chart review of 1,162 patients who underwent penetrating keratoplasty at Wills Eye Institute, Philadelphia, PA, USA, from 2001 to 2005. Their reported frequency of 10.8% is just above half of the 19% proportion we identified during the same period. This discrepancy can be explained by their leading indication for penetrating keratoplasty, which was pseudophakic corneal edema, accounting for 28.4% of their cases. Furthermore, the observed differences may also be attributed to ethnic background, with a higher proportion noted among individuals of European ancestry, as well as demographic and methodological factors [34].

The proportion of Fuchs’ dystrophy cases reported by Keane et al. [25], who analyzed over 40,000 corneal grafts performed from 1987 to 2020 and registered with the Australian Corneal Graft Registry (ACGR), at 18%, is comparable to our findings of 24% in Freiburg in 1985–2015 (Table 4). However, it is not known which diagnoses made by ophthalmologists or optometrists were verified histopathologically. The contribution of Fuchs’ dystrophy to corneal lesions observed in New Zealand are less than half of our findings, likely due to the significant contribution of keratoconus to their total corneal cases: Edwards et al. [26] analysed data on penetrating and lamellar keratoplasty from 1,370 corneal grafts, collected by the New Zealand National Eye Bank in Auckland from 1991 to 1999. They reported a proportion of 4.4% for Fuchs’ dystrophy, compared to our 9.2% (Table 4). Similarly, Cunningham et al. [27] examined records of 2,205 corneal transplants from the New Zealand National Eye Bank over the decade from 2000 to 2009. Their findings of 8.2% contrast with our 19% observed in Freiburg during the same period.

Matthaei et al. [29] reported that Fuchs’ dystrophy accounted for 10.2% of all corneal cases in Europe, a figure that is lower than the findings in Manchester in 2007–2008 [22], at 15.4%, and it constitutes only half of the 20% proportion we histologically confirmed in Freiburg (Table 4). Their study evaluated 141 publications from 37 countries, documenting 180,865 cases of penetrating keratoplasty from 1980 to 2014. In contrast, the relative frequency of 12.9% observed in North America aligns well with data published from Atlanta (16%) for the period 1941–1995 [10] and from Philadelphia (10.8%) for 2001–2005 [23].

Rohrbach et al. [8] documented a rising trend in the proportion of *keratoconus* cases over the years 1960, 1980, and 1990 in Tübingen (Table 4), similar to the pattern we observed in Freiburg. While the percentage of keratoconus cases in Manchester, at 21.7% during 2007–2008 [22], and in Philadelphia, at 16% during 2001–2005 [23], closely aligns with our data from Freiburg (19% and 18%, respectively), a lower proportion of 11% is reported in Atlanta [10] compared to Freiburg’s 19% during their study period from 1941 to 1990. The lower relative frequency in Atlanta might again be due to inclusion of corneal diagnoses of enucleated eyes, which to our own experience rarely contain a keratoconus.

Kelly et al. [24] conducted a cohort study involving 4,834 eyes of 4,060 patients, as recorded by the Australian Corneal Graft Registry (ACGR) from 1985 until 2009. In our study, the proportion of keratoconus cases in Freiburg was 19% from 1985 to 2009 and 15% from 1985 to 2015 (Table 4). These figures represent only two-thirds of the proportions reported for Australia by Kelly et al. [24] in 1985–2009 and Keane et al. [25] in 1985–2020, which were 31% and 24%, respectively. Moreover, New Zealand exhibits an exceptionally high percentage of keratoconus, with rates of 45.6% from 1991 to 1999 and 41.1% from 2000 to 2009. These rates are approximately double our own results of 22% and 18%, respectively, within the same time intervals. The higher prevalence of keratoconus among Maori and Polynesians, compared to Caucasians, results in New Zealand reporting the highest proportion of keratoplasty for keratoconus worldwide [35].

In contrast, a study from North China conducted by Xie et al. [28] reported that 12.9% of cases were attributed to keratoconus (Table 4), a figure slightly exceeding half of the proportion observed in Freiburg (23%) during the same period. This study involved a retrospective review of records from 1,702 patients who underwent penetrating keratoplasty at Shandong Eye Institute, China, between 1997 and 2002.

According to Matthaei et al. [29] keratoconus accounted for 24.2% of all corneal cases in Europe from 1980 to 2014, based on 51 studies (Table 4). This figure is 50% higher than the percentage of 16% we observed in Freiburg. The relative frequency of 14.2% for keratoconus in North America, derived from 29 studies, aligns well with data from Atlanta (11%) [10] and Philadelphia (19%) [23]. Furthermore, the percentage of 8.1% for keratoconus in Asia, based on 21 studies [29], is lower than that reported by Xie et al. [28] for North China (12.9%).

According to Rahman et al. [22], keratitis accounted for 5.8% of all corneal cases in Manchester during 2007–2008, encompassing only ulcerative and infectious cases. This focus may explain the discrepancy with the 12% rate observed in Freiburg (Table 4). The proportion of keratitis cases observed in Freiburg from 1945 to 1995 (14%) is comparable to that reported in Atlanta (19%), where all corneal diagnoses were included, even those from enucleated or eviscerated eyes [10]. In contrast, our findings between 2001 and 2005 show a proportion of 17%, which differs significantly from the 2.7% reported in Philadelphia during the same period, where the focus was solely on viral keratitis [23].

From 1985 to 2015, the relative frequency of keratitis cases in Freiburg was 12%. This figure is three times higher than the 3.7% reported in Australia over a comparable period (1985–2020), as documented by Keane et al. [25], where diagnoses were based on histologically unconfirmed clinical assessments (Table 4). Two studies from New Zealand reported proportions of keratitis at 7.3%, encompassing herpes simplex and herpes zoster keratitis, from 1991 to 1999 [26], and 7.9% from 2000 to 2009 [27]. These figures are almost half of those observed in Freiburg, which were 12% and 15% during the same respective periods, which may be due to different inclusion criteria.

Matthaei et al. [29] reported that keratitis accounted for 13.2% of cases in Europe from 1980 to 2014, a figure that aligns closely with our findings in Freiburg, which showed 13% (Table 4). For Asia, the authors noted that keratitis contributed to 32.5% of all corneal cases, consistent with the results of Xie et al. [28], who documented a proportion of 31.8% for North China from 1997 to 2002. This proportion was more than twice as high as our findings in Freiburg, which stood at 15% during the same period.

In our study, *transplant failures* accounted for nearly the same proportion of corneal diagnoses, at 11%, as keratitis. During 2007–2008, our findings in Freiburg show a proportion of 17%, which is lower than the 22.3% reported from Manchester during the same period [22] (Table 4). Additionally, our figure of 10% from 2001 to 2005 is lower than the 22% observed in Philadelphia [23]. In Australia, Kelly et al. [24] reported that transplant failures accounted for 19% of all corneal cases from 1985 to 2009, compared to 11% in Freiburg. Keane et al. [25] found that such failures constituted 24% of cases from 1985 to 2020, whereas our study identified a rate of 13% in Freiburg from 1985 to 2015. We hypothesize that our lower rate may be attributed to a more rigorous post-operative anti-inflammatory regimen, including consistent long-term administration of low-dose topical steroids and systemic immunosuppression in high-risk keratoplasty cases.

The proportions of transplant failures reported in New Zealand by Edwards et al. [26], at 8.7% from 1991 to 1999, and by Cunningham et al. [27], at 17.0% from 2000 to 2009, align well with our own data from Freiburg during the same periods, which were 7.1% and 13%, respectively (Table 4). According to Xie et al. [28], transplant failures accounted for 4.5% of corneal cases in North China from 1997 to 2002, which aligns precisely with our own findings of 4.5% during the same period.

Matthaei et al. [29] found that transplant failures accounted for 16.3% of all corneal cases in North America from 1985 to 2014 (Table 4), whereas this proportion was lower in Asia, at 11.1%. Although they do not provide data for Europe, our findings of 13% in Freiburg are quite comparable.

### Conjunctiva

In our study, *pterygium* was identified as the most common conjunctival diagnosis, constituting 29% of all cases. We observed a significant upward trend in the relative frequency of pterygia over the last three analysed periods, with rates climbing from 4.6% in 1975–1984 to 37% in 2005–2015 (Table 1). Notably, the number of pterygia cases surged from 7 specimens in 1988 to 127 in 2015 (Fig. 3). This trend can be attributed, in part, to the implementation of histological examinations for all pterygia cases following Heinrich Witschel’s appointment as director in 1988. Furthermore, the emphasis on corneal and conjunctival diseases under the directorship of Reinhard, beginning in 2003, contributed to an increase in the number of conjunctival specimens examined.

Given that the pathogenesis of pterygia is strongly linked to UV exposure, the observed rise may also be attributed to the general increase in UV exposure in our region. However, the relationship between UV exposure and pterygium is complex and multifactorial. Increased outdoor activities (“surfer’s eye”) and occupations (farming, construction, military service), along with environmental influences like air pollution, are reinforced by demographic factors including age, male gender, rural residence, and lower education levels, all contributing to this pattern. Interestingly, the proportion of pinguecula cases remained consistently low, ranging from 2.4% to 4.2%, with no noticeable increase in the final study interval. This observation is consistent with findings from an epidemiological study in India, which reported a significant correlation between higher UV exposure and pterygium, but not pinguecula [36].

There has been a notable expansion in the age range of patients diagnosed with pterygium over the course of our study, extending from 10 to 92 years. In the interval from 1955 to 1964, the youngest patient was 31 years old and the oldest was 41 years old. In contrast, during the most recent interval, patient ages spanned from 4 to 96 years (Table 2). This broadening age range may indicate increased UV exposure across all age groups, in addition to earlier interventions facilitated by improved surgical techniques and an aging population. Figure 7 demonstrates that beyond age 17, the relative frequency of pterygium increases and reaches its highest levels between the ages of 46 and 76.

*Conjunctival nevus* accounted for 12% of all cases throughout our observation period. It was the most prevalent conjunctival diagnosis during the periods 1965–1974 (16%) and 1975–1984 (20%) (Table 1). However, the subsequent significant rise in pterygia cases resulted in it overtaking conjunctival nevus as the leading diagnosis. Conjunctival nevi are common benign pigmented lesions of the eye, and their growth can be influenced by several factors. These include genetic predisposition, prolonged sun exposure, and young age. Consequently, the age range of patients with conjunctival nevi expanded from 25 years in the first interval (1945–1954) to 91 years in the period from 1995 to 2004 (Table 2). Notably, we observed an increase in the number of surgically excised nevi starting from age 6, with a peak incidence at age 14 (Fig. 7).

In our study, *pyogenic granuloma* with a relative frequency of 7.0%, was the third most common conjunctival diagnosis. The development of pyogenic granulomas is thought to involve an abnormal response of the blood vessels to various triggers, such as trauma, injury or infection. These raised, red lesions can also form as a postoperative complication following conjunctival or eyelid surgery with conjunctival involvement. While their numbers did not rise above two, three and seven cases during the first, second and third intervals, we observed a subsequent steady increase to 194 (6.8%) cases during 2005–2015 (Table 1).

In the period from 1945 to 1954, the age range of patients with pyogenic granuloma spanned from 4 to 12 years (Table 2). By 2005 to 2015, this range had expanded significantly from a span of just 8 years to an impressive 90 years, with the youngest patient being 2 years old and the oldest 92 years old. A similar trend is evident for *conjunctival cysts*, fluid-filled epithelial sacs on the conjunctiva. Initially, from 1945 to 1954, the youngest patient was 14 years old, decreasing to just 1 year old by the end of the study. Concurrently, the age of the oldest patient increased from 42 to 85 years. Thus, the age range for patients with cysts broadened considerably from 28 years to 84 years over the study period.

This concurrent expansion in age ranges might be attributed to an increase in postoperative cases among older patients, who developed pyogenic granuloma or conjunctival cysts as complications following conjunctival and lid surgeries. Additionally, the overall rise in the number of conjunctival and lid surgeries could contribute to the increased relative and absolute frequencies of pyogenic granulomas. The slight increase in the proportion of conjunctival cysts to 10% during the period from 1965 to 1974 (Table 1) may be attributed to Mackensen and his introduction of ocular surgery under the microscope, which led to a subsequent increase in conjunctival and eyelid surgeries.

*Conjunctivitis* constituting 6.2% of all conjunctival cases was diagnosed more frequently during the intervals from 1945 to 1964, accounting for 21% and 20% of cases, respectively (Table 1). Subsequently, there was a significant decline in its diagnosis, with relative frequencies decreasing from 9.4% between 1965 and 1974 to just 4.2% in the final interval. This trend may reflect advancements in topical anti-inflammatory [37] and antibiotic medications [38], which have likely reduced the need for biopsies to cases that are non-responsive to treatment, thereby helping to rule out malignancies.

Figure 7 illustrates that conjunctivitis can affect individuals across all age groups. Reflecting this, the age range of our patients expanded from 58 years (17 to 75 years) in the period from 1945 to 1954, to 91 years (0 to 91 years) in the interval from 2005 to 2015 (Table 2). Conjunctivitis is particularly common in children, largely due to the close contact environments of kindergartens and schools, where it is often caused by viruses or bacteria. Adults, in addition, may encounter conjunctivitis due to exposure to irritants, allergens, or through contact lens use. Although less common in the elderly, conjunctivitis can still occur in this age group due to changes in the immune system or conditions such as dry eye syndrome.

*CIN* accounted for 5.0% of all conjunctival diagnoses, consistently occupying lower positions within the “Top 10” rankings across each interval. Its relative frequency varied between 3.1% and 5.3% from the second interval (1955–1965) to the sixth interval (1995–2004), ultimately rising to 6.6% in the final period from 2005 to 2015 (Table 1). This aligns with the findings of epidemiological studies conducted in the UK, which have observed no significant change in the prevalence of ocular surface squamous neoplasia (OSSN) since 1996 [39]. Regarding the increase in the number and relative frequency of CIN diagnoses in our most recent interval, it is notable that two-thirds of all CIN biopsies were excised during Reinhard’s directorship.

One of the primary risk factors for the development of CIN is prolonged exposure to UV radiation from sunlight. Additional risk factors include human papillomavirus (HPV) infection, long-standing irritation or chronic inflammation of the conjunctiva, and genetic predisposition. Given that CIN is more commonly diagnosed in older adults, it appears that cumulative exposure to these risk factors over time contributes significantly to its development. This trend is illustrated in Fig. 7, which shows that the first cases of CIN occur in patients as young as 20 years old, with a slight increase in frequency observed up to the age of 69, before declining up to age 91.

We now compare the relative frequencies of the five most common conjunctival conditions identified in our study with those described in the previously cited works of Grossniklaus et al. [16], Spraul and Grossniklaus [10], Shields et al. [17] and Domingo et al. [14] (Table 5). Additionally, Pellerano et al. [40] conducted a retrospective, non-interventional case series involving 138 consecutive patients with conjunctival masses evaluated at two tertiary referral centres in Santo Domingo, Dominican Republic, between 2010 and 2018. Alkatan et al. [41] analysed all consecutive conjunctival tissue specimens submitted for histopathological assessment to the Pathology Department at the College of Medicine, King Saud University, Riyadh, Saudi Arabia, from 2015 to 2019. Mondal et al. [42] examined 120 conjunctival biopsy samples from 117 patients received at the Department of Pathology, Medical College, Kolkata, West Bengal, India, between 2003 and 2010.

**Table 5:** Comparison of conjunctival diagnoses with results from other studies

Study	Region	Time period	Pterygium (%)	Nevus (%)	Pyogenic granuloma (%)	Conjunctivitis (%)	CIN (%)
Grossniklaus et al. [16]	Baltimore (US)	1923–1984	18	8.1	4.2	10.2^a^	8.7^b^
*Our study*	Freiburg (DE)	1945–1984	5.3	17	6.5	10	4.1
Spraul and Grossniklaus [10]	Atlanta (US)	1941–1995	13	9.3	1.3	21^c^	8.2
*Our study*	Freiburg (DE)	1945–1995	13	17	8.4	9.1	3.8
Shields et al. [17]	Philadelphia (US)	1974–2015	2.0^d^	23^d^	1.3^d^	4.2^d,e^	5.5^d^
*Our study*	Freiburg (DE)	1974–2015	6.0	13	2.9	6.0	5.4
Pellerano et al. [40]^f^	Dominican Republic	2010–2018	-	35	-	2.9	12
*Our study*	Freiburg (DE)	2010–2015		10		4.8	7.2
Alkatan et al. [41]	Saudi Arabia	2015–2019	40	13	6.4	2.7^g^	1.8
*Our study*	Freiburg (DE)	2010–2015	41	10	7.2	4.8	7.2
Mondal et al. [42]	Eastern India	2003–2010	22.5	4.16	8.33	3.33^g^	10.83
*Our study*	Freiburg (DE)	2003–2010	39	11	6.8	4.3	5.8
Domingo et al. [14]	Philippines	2003–2012	-	9.4	5.5	3.9^h^	10.2
*Our study*	Freiburg (DE)	2003–2012		11	7.0	4.6	6.3

This table presents a comparison of the relative frequencies of the five most prevalent conjunctival diagnoses in our study with those reported in existing literature

^a^ Nonspecific non-granulomatous inflammation, nonspecific granulomatous inflammation, lymphoid hyperplasia, vernal inflammation, inflammatory pseudotumor

^b^ Dysplasia, carcinoma in situ

^c^ Non-granulomatous inflammation, granulomatous inflammation, lymphoid hyperplasia, (giant) papillary conjunctivitis, sarcoidosis, allergic conjunctivitis

^d^ Proportion of 5,002 cases with clinically suspected neoplasia

^e^ Conjunctivitis, reactive lymphoid hyperplasia

^f^ This study includes conjunctival tumours only

^g^ Reactive lymphoid hyperplasia

^h^ Atypical lymphoid hyperplasia, reactive lymphoid hyperplasia

Situated at 48° N, Freiburg lies approximately 980 kilometres closer to the pole—and thus further from the equator—than Baltimore, which is located at 39.3° N. One might initially attribute the higher frequency of *pterygia* in Baltimore (18% between 1923 and 1984) [16] compared to Freiburg (5.3% between 1945 and 1984) to reduced UV exposure among the Freiburg population (Table 5). However, this hypothesis is challenged by data from Atlanta, positioned at 33.8° N and some 1,600 kilometres nearer the equator than Freiburg, yet reporting an identical proportion of pterygia diagnoses (13% between 1941 and 1995) [10] to that observed in Freiburg (13% between 1945 and 1995). However, this discrepancy may be attributed to a higher proportion of conjunctivitis cases by inclusion of enucleated bulbi in the Atlanta cohort, potentially skewing the data. In contrast, Shields et al. [17] reported a notably lower proportion of pterygia cases in Philadelphia—just 2% compared to our 6%—for the period 1974–2015. This discrepancy is likely attributable to their database comprising primarily patients referred to an ocular oncology service, which inherently results in an overrepresentation of suspicious and malignant lesions.

This referral bias may also account for Shields et al.’s finding that *nevi* were the most common conjunctival diagnosis (23%), whereas in our study, nevi accounted for only 12% of cases during the same interval (1974–2015) (Table 5). However, our study identified a higher relative frequency of nevi—17% in both 1945–1984 and 1945–1995—compared to the 8.1% reported in Baltimore (1923–1984) [16] and 9.3% in Atlanta (1941–1995) [10]. These differences may reflect the lower proportion of conjunctival nevi in non-Caucasian populations [17], which were likely more strongly represented in the Baltimore, Atlanta, and West Indian studies—where only 4.16% of nevi were reported between 2003 and 2010 [42]—compared to our predominantly Caucasian study population. In contrast, the remarkably high relative frequency of conjunctival nevi (35%) reported by Pellerano et al. in the Dominican Republic between 2010 and 2018 [40] may be explained by the demographic composition of their study: half of the participants were children and adolescents, with nevi occurring in 50% of specimens from children and in 33% of those from adolescents. The proportion of conjunctival nevi reported by Domingo et al. (9.4% between 2003 and 2012) [14] aligns well with the relative frequency observed in Freiburg during the same period (11%), based on histological diagnoses.

The markedly high proportion of *pterygia* observed in Freiburg—41% (2010–2015) and 39% (2003–2010)—stands out, especially given the city’s distance from the equator (2,592 km farther than Riyadh), compared to 40% in Saudi Arabia [41] and 22.5% in Eastern India [42] during the respective periods (Table 5). Such findings may indicate a referral bias, potentially linked to the expanded catchment area of the Freiburg Eye Center during Reinhard’s directorship. While these data suggest that geographic location alone does not drive diagnostic trends in this cohort, further analysis of regional variations remains a compelling avenue for future investigation.

The relative frequency of *pyogenic granuloma* cases is reported in six of the studies listed in Table 5: 4.2% in Baltimore (1923–1984) [16], 1.3% in Atlanta (1941–1995) [10], 1.3% in Philadelphia (1974–2015) [17], 6.4% in Saudi-Arabia (2015–2019) [41], 8.33% in Eastern India (2003–2010) [42], and 5.5% in the Philippines (2003–2012) [14]. With one exception, these figures are similar to our findings in Freiburg of 6.5% (1945–1984), 8.4% (1945–1995), 2.9% (1974–2015), 7.2% (2010–2015), 6.8% (2003–2010) and 7.0% (2003–2012). Strikingly, the relative frequency of pyogenic granulomas in Atlanta was 6.5 times lower than that observed in our study for the period from 1945 to 1995 [10]. This discrepancy may, at least in part, be explained by the inclusion of enucleated bulbi in the Atlanta dataset, which may have artificially elevated the proportion of conjunctivitis diagnoses.

The proportion of *conjunctivitis* diagnoses among all conjunctival cases in Freiburg, ranging from 4.3% to 10% across the respective study periods, closely aligns with the results reported in the studies from Baltimore [16], Philadelphia [17], the Dominican Republic [40], Saudi Arabia [41], Eastern India [42] and the Philippines [14] (Table 5). In contrast, Atlanta reported a relative frequency of conjunctivitis cases more than twice as high (21% between 1941 and 1995) compared to our own findings (9.1%)—a discrepancy that may again be attributable to the inclusion of enucleated bulbi in Atlanta [10].

Diagnoses of *CIN* are consistently more prevalent in populations with higher solar exposure, as evidenced by studies from Baltimore (8.7% vs. Freiburg’s 4.1%) [16], Atlanta (8.2% vs. Freiburg’s 3.8%) [10], Philadelphia (5.5% vs. Freiburg’s 5.4%) [17], the Dominican Republic (12% vs. Freiburg’s 7.2%) [40], Eastern India (10.83% vs. Freiburg’s 5.8%) [42] and the Philippines (10.2% vs. Freiburg’s 6.3%) [14] (Table 5). In Philadelphia, the focus on melanocytic conjunctival tumours likely inflated the proportion of nevi, obscuring differences in CIN rates. Conversely, in Saudi Arabia—where darker skin pigmentation is common—cultural practices such as protective clothing may mitigate ocular UV exposure. A lower prevalence of HPV infection could also explain the notably reduced CIN rate of 1.8% (compared to Freiburg’s 7.2%) [41].

### Orbit

The orbit’s complex anatomy—deep-seated and encased by bone, with critical neurovascular structures—makes biopsies and surgical excisions technically demanding. These procedures carry a heightened risk of complications, including potential damage to the optic nerve or extraocular muscles. Primary orbital diseases (e.g., tumours, inflammatory conditions) are relatively rare compared to conditions affecting the eyelid (e.g., BCC), cornea (e.g., infections, dystrophies), or conjunctiva (e.g., conjunctivitis, pinguecula). The lower occurrence of orbital diseases naturally results in fewer specimens and their modest rise across our study period (Table 1) (Fig. 4). The observed 40% decrease—from 144 specimens (1995–2004) to 85 specimens (2005–2015)—may reflect the enhanced quality of advanced imaging techniques, such as MRI and CT. These methods enable more accurate, non-invasive diagnosis of orbital diseases, thereby reducing the necessity for invasive biopsies except in essential cases.

Most orbital lymphomas are non-Hodgkin lymphomas (NHL), particularly extranodal marginal zone B-cell lymphomas (EMZL), which are strongly associated with aging. The incidence of NHL rises significantly after age 60, peaking—consistent with our findings—in the seventh decade of life [43] (Fig. 8). Orbital inflammation, by contrast, typically presents earlier due to autoimmune triggers or acute inflammatory processes. The age distribution of orbital metastases mirrors that of their primary cancers; for example, breast or lung cancer metastases often occur in middle age (40s–60s). Notably, no hemangioma cases were diagnosed before age 29 in our study (Fig. 8). Unlike capillary hemangiomas, which usually appear in infancy or early childhood and often regress spontaneously, cavernous hemangiomas are slow-growing, benign vascular malformations. These lesions frequently remain asymptomatic for years or even decades, obviating the need for biopsy or surgical intervention during this period [44].

*Orbital* *inflammation* is an umbrella term that encompasses a wide range of non-infectious, non-neoplastic inflammatory conditions (e.g., thyroid eye disease, sarcoidosis, myositis and nonspecific orbital inflammation). This broad classification naturally increases its reported relative frequency of 12% within our study compared to rarer, more specific orbital diseases like primary tumours or vascular malformations.

The marked increase in *orbital lymphoma* diagnoses after 1994—culminating in its emergence as the most common orbital pathology in our two most recent study intervals (accounting for 17% and 28% of all orbital specimens) (Table 1)—appears to reflect a genuine rise in incidence rather than merely improved diagnostic accuracy. Although advances in immunohistochemistry and molecular pathology have sharpened our ability to differentiate lymphomas from inflammatory lesions including reactive lymphoid hyperplasia, the consistent relative frequency of inflammatory cases—ranging between 9% and 14% across most intervals (with the sole outlier of 42% in 1955–1964)—implies that reclassification alone does not explain this pattern. Instead, it points to a real increase in the occurrence of orbital lymphomas over time. This trend aligns with the rising incidence of *ocular adnexal lymphoma* observed in Denmark—where rates increased from 0.086 to 0.307 per 100,000 between 1980 and 1984 and 2013–2017 [45]—and with the increase in orbital lymphoma reported in South Korea from 1999 to 2016 [46]. A large American epidemiological study of 17,878 patients also demonstrated an increase in ocular and orbital lymphoma between 1995 and 2003, although a subsequent decline was noted from 2003 to 2018 [47].

The diagnosis of prolapsed *orbital fat* first appeared among the “Top 10” orbital pathologies during the 1985–1994 interval (Table 1), with a relatively stable and slightly increasing frequency (ranging from 9% to 13% in subsequent periods). This pattern suggests that surgical intervention for transconjunctival herniation of extraconal fat—secondary to Tenon’s capsule dehiscence—has been routinely performed at our institution only since 1985. Given its clinical resemblance to adipocytic neoplasms [48], all excised specimens undergo histological examination as a standard precaution.

Between 1995 and 2004, *orbital metastases* accounted for 17 diagnoses (12% of cases), making them the second most common orbital pathology. The 1990s and early 2000s saw the routine adoption of high-resolution MRI and CT scans in clinical practice. These imaging modalities significantly improved the detection of small or subtle orbital metastases, which might have been missed or misdiagnosed in earlier decades.

The peak in diagnosed *orbital hemangiomas* (benign vascular malformations), which accounted for 10% of cases (9/91) between 1975 and 1984 (Table 1), followed by a subsequent decline to 2.4% of cases (2/85) between 2005 and 2015, likely reflects the introduction and refinement of CT scanning during the 1970s, which improved detection of these previously perhaps underdiagnosed or misclassified lesions. The later adoption of MRI in the 1980s, while offering superior visualisation, enabled more precise differentiation between hemangiomas and other orbital lesions (such as lymphangiomas or cavernous malformations), potentially reducing the number of cases classified as “hemangioma”. Correctly diagnosed hemangiomas are removed only in cases of optic nerve compression and/or severe ocular motility impairment.

Table 6 compares the relative frequencies of the five most common orbital conditions identified in our study with the previously cited findings of Spraul and Grossniklaus [10], as well as those reported in other relevant studies. Bonavolontà et al. [49] analysed 2,480 space-occupying orbital lesions (1976–2011) at the Orbital Unit, University of Naples Federico II, Italy, using clinical history, ocular examination, diagnostic imaging, and histopathologic analysis where available. Koukkouli et al. [50] retrospectively studied 112 orbital biopsies from 101 patients (2003–2015) at the Department of Ophthalmology, St. James University Hospital, Leeds, UK. Kennedy [18] reviewed 820 orbital cases diagnosed between 1949 and 1983 at the Department of Ophthalmology, University of Rochester School of Medicine and Dentistry, NY, USA. Shields et al. [19] examined 654 consecutive orbital lesion biopsies (1962–1982) at Wills Eye Hospital, Philadelphia, PA, USA. In a retrospective, clinicopathologic case series, Kneafsey et al. [51] reviewed 83 orbital biopsies performed in 77 patients at the Mater Misericordiae University Hospital (MMUH), Dublin, Ireland, between January 2008 and January 2018. After excluding 11 lacrimal gland samples, we compared their findings with our own study results. Shinder et al. [52] retrospectively assessed the medical records of 268 patients with orbital masses (1998–2009) at the Orbital Oncology Service, Section of Ophthalmology, Department of Head and Neck Surgery, The University of Texas M. D. Anderson Cancer Center, Houston, TX, USA. Shikishima et al. [53] retrospectively reviewed 104 patients with orbital tumours (1983–2002) at the Department of Ophthalmology, Jikei University School of Medicine, Tokyo, Japan.

**Table 6:** Comparison of orbital and lacrimal gland diagnoses with results from other studies

		Orbital diagnoses						Lacrimal gland diagnoses		
Study	Region	Time period	Inflammation (%)	Lymphoma (%)	Fat tissue (%)	Metastasis (%)	Hemangioma (%)	Time period	Dacryoadenitis (%)	Pleomorphic adenoma (%)
Bonavolontà et al. [49]	Naples (IT)	1976–2011	13	12	-	3.0	12	1976–2011	27	32
*Our study*	Freiburg (DE)	1976–2011	9.3	10	8.1	5.8	6.3	1975–2015	39	15
Koukkouli et al. [50]	Leeds (UK)	2003–2015	30	19	-	3.7	6.5	2003–2015	50	25
*Our study*	Freiburg (DE)	2003–2015	4.6	18	6.4	2.9	3.5	2005–2015	11	4.4
Kneaufsey et al. [51]	Dublin (IR)	2008–01/2018	36	12	-	6.5	10	2008–01/2018	18	-
*Our study*	Freiburg (DE)	2008–2015	4.2	15	0.8	4.2	0.0	2005–2015	11	4.4
Spraul and Grossniklaus [10]	Atlanta (US)	1941–1995	15	6.6	-	4.6	4.6	1941–1995	57	3.1
*Our study*	Freiburg (DE)	1945–1995	14	1.2	4.3	4.3	8.0	1945–1994	23	24
Kennedy [18]^a^	Rochester (US)	1949–1983	12	12^b^	-	3.5	4.6	1949–1983	27	27
*Our study*	Freiburg (DE)	1949–1983	17	1.0	0.5	4.1	8.7	1945–1984	23	21
Shields et al. [19]	Philadelphia (US)	1962–1982	21	5.9	-	3.9	6.6	1962–1982	54	18
*Our study*	Freiburg (DE)	1962–1982	13	1.4	0.0	3.4	8.3	1965–1984	28	25
Shinder et al. [52]	Houston (US)	1998–2009	15	24	-	8.1^c^	2.7^d^	1998–2009	-	7.4
*Our study*	Freiburg (DE)	1998–2009	6.6	21	12	8.6	4.6	1995–2004	32	7.3
Shikishima et al. [53]^e^	Tokyo (JP)	1983–2002	22	12	-	3.0	5.9	1983–2002	3.8	64
*Our study*	Freiburg (DE)	1983–2002	9.6	7.2	9.6	9.2	6.0	1985–2004	45	18

This table presents a comparison of the relative frequencies of the five most prevalent orbital diagnoses in our study with those reported in existing literature.

^a^ Of the 820 clinical diagnoses, 500 were histologically confirmed, while the remainder were considered diagnostically convincing based on imaging findings

^b^ Lymphoma, lymphosarcoma, Burkitt’s lymphoma, multiple myeloma, Hodgkin`s disease

^c^ Of the 28 clinical diagnoses, 15 were histologically confirmed

^d^ Cavernous hemangioma; of the 15 clinical diagnoses, 5 were histologically confirmed

^e^ Summary of in-house cases (1983–2002) and those reported in Japanese literature (1980–2004)

The relative frequencies of *orbital inflammation* in studies from Naples (13% vs. 9.3% in our series, 1976–2011) [49], Atlanta (15% vs. 14%, 1945–1995) [10], and Rochester (12% vs. 17%, 1949–1983) [18] align closely with our findings in Freiburg. In contrast, the results from Leeds (30% vs. 4.6%, 2003–2015) [50], Dublin (36% vs. 4.2%, 2008–2015) [51], Philadelphia (21% vs. 13%, 1962–1982) [19], Houston (15% vs. 6.6%, 1998–2009) [52], and Tokyo (22% vs. 9.6%, 1983–2002) [53] show marked divergence. These centres report proportions of orbital inflammation that are 6.5-, 8.6-, 1.5-, 2.3-, and 2.3-fold higher, respectively, than those observed in our cohort (Table 6). Leeds Teaching Hospitals, a major tertiary referral centre for Leeds, Yorkshire, and Northern England, particularly for complex and inflammatory eye conditions, may exhibit a referral bias due to its specialised caseload and strong research focus on orbital diseases. This likely accounts for the high proportion (30%) of orbital inflammation cases reported in their series. Similarly, the MMUH in Dublin, serving as a tertiary referral hub for orbital inflammatory diseases in the North Leinster region, may also reflect such a bias, explaining the 36% contribution of inflammation to orbital pathologies. During the 1962–1982 period, our Freiburg data showed a lower proportion of orbital inflammation (9.2% in 1965–1974) (Table 1) compared to Philadelphia (21%), likely because other diagnoses—such as fibrotic and normal muscle (each 11%) and muscular dystrophy (9.2%)—were more prominent. Similarly, in the 1995–2004 and 2005–2015 intervals, our rates of orbital inflammation remained below those reported in Houston (15%), primarily due to the rising contributions of lymphoma (17% and 28%), metastasis (12% and 5.9%), and orbital fat tissue removal (10% and 13%). This pattern also accounts for the more than 50% lower relative frequencies observed in our series (9.6%) compared to the Tokyo study (22%).

The proportions of *orbital lymphoma* in our Freiburg series closely correspond to those reported from Naples (12% vs. 10% in our series, 1976–2001) [49], Leeds (19% vs. 18%, 2003–2015) [50], Dublin (12% vs. 15%, 2008–2015) [51] and Houston (24% vs. 21%, 1998–2009) [52] (Table 6). By contrast, studies conducted before 1995—such as those from Atlanta (6.6% vs. 1.2%, 1945–1995) [10], Rochester (10% vs. 1.0%, 1949–1983) [18], and Philadelphia (5.9% vs. 1.4%, 1962–1982) [19]—show markedly higher relative frequencies. This discrepancy can be attributed to the rarity of lymphoma cases in our series prior to 1995, with only three cases documented since 1945. Multiple epidemiological studies have documented a marked and sustained increase in NHL incidence in the USA from the 1950s through the 1990s, with annual increases of 3–4%—a trend that was more pronounced than in most European countries, including Germany [54–56]. This rise was especially notable for extranodal lymphomas, which include orbital lymphomas [54, 55]. A similar, though less pronounced, discrepancy is seen in the Tokyo study (12% vs. 7.2%, 1983–2002) [53], likely because its timeframe overlaps with our 1995–2004 interval—a period during which the proportion of orbital lymphomas in Freiburg began to rise, reaching 17% (Table 6).

None of the aforementioned comparative studies reported *orbital fat tissue* removal, and we are unaware of any published data on its relative frequency in the general population or across all orbital pathologies. The slight increase in orbital samples following Witschel’s appointment as director of the Freiburg Eye Hospital in 1988 is mirrored in the proportions of orbital fat tissue calculated for the individual time intervals of the studies considered for all other orbital diagnoses (Table 6).

The proportion of *metastasis* among all orbital pathologies in Freiburg closely align with those reported in the Naples (3.0% vs. 5.8% in our series, 1976–2001) [49], Leeds (3.7% vs. 2.9%, 2003–2015) [50], Dublin (6.5% vs. 4.2%, 2008–2015) [51], Atlanta (4.6% vs. 4.3%, 1945–1995) [10], Rochester (3.5% vs. 4.1%, 1949–1983) [18], Philadelphia (3.9% vs. 3.4%, 1962–1982) [19] and Houston (8.1% vs. 8.6%, 1998–2009) [52] studies (Table 6). A notable discrepancy exists between the relative frequencies reported from Freiburg (9.2%) and Tokyo (3%) in the 1983–2002 period. In Germany and other Western countries, breast and lung carcinomas are the most frequent sources of orbital metastases, reflecting the higher incidence of these cancers in the population. In contrast, Japanese series show a relatively lower rate of orbital metastases, with a different distribution of primary tumours—such as a higher proportion of liver and stomach cancers, which are less likely to metastasise to the orbit compared to breast and lung cancers [57]. Additionally, the overall incidence of metastatic orbital tumours in Japan is lower, as demonstrated by large case series and literature reviews, which consistently report fewer cases of orbital metastases relative to Western populations [53, 57].

The Orbital Unit at the University of Naples Federico II, as a major referral centre for vascular orbital lesions, including cavernous *hemangiomas*, likely accounts for the higher relative frequency of these orbital pathologies in their series (12% vs. 6.3% in our series, 1976–2011) [49] (Table 6), a discrepancy attributable to referral bias. A similar pattern is evident in the results from the Leeds (6.5% vs. 3.5%, 2003–2015) [50] and Dublin (10% vs. 0.0%, 2008–2015) [51] tertiary referral centres. In contrast, our findings align closely with those from Tokyo (5.9% vs. 6.0%, 1983–2002) [53]. Compared to the four US studies—Atlanta (4.6% vs. 8.0% in our series, 1945–1995) [10], Rochester (4.6% vs. 8.7%, 1949–1983) [18], Philadelphia (6.6% vs. 8.3%, 1962–1982) [19] and Houston (2.7% vs. 4.6%, 1998–2009) [52]—our data consistently reveal higher proportions of hemangiomas across all corresponding time intervals. This discrepancy likely reflects the earlier and more widespread adoption of advanced imaging in the USA, where a preference for conservative management of benign lesions has reduced the reliance on histological diagnosis [58].

### Lacrimal gland

Because the orbit provides structural support and protection for the lacrimal gland—which in turn ensures ocular lubrication and health—we address the lacrimal gland immediately after discussing the orbit. In Freiburg, the combined proportion of lacrimal gland pathologies attributed to *pleomorphic adenoma* and *dacryoadenitis* showed a marked proportion since 1965: from 50.3% (42% and 8.3%, respectively; 1965–1974) to 55% (17% and 38%; 1975–1984), then to 53% (32% and 21%; 1985–1996), peaking at 68% (11% and 57%; 1995–2004) before declining to 15.4% (4.4% and 11%; 2005–2015) (Table 1). The initial rise in the late 1960s can be attributed to Mackensen’s introduction of microsurgical techniques. Enhanced histopathologic recognition of these entities as distinct lacrimal gland conditions, combined with the advent of CT and MRI in the 1970s and 1980s, further facilitated their detection. However, in the most recent period, the growing frequency of other lacrimal gland diagnoses—such as normal findings, cysts, and lymphomas—led to a relative decline in the representation of both dacryoadenitis and pleomorphic adenoma.

Given the low number of specimens beyond the orbit, annual analyses of diagnosis frequencies and proportions were not conducted. To allow direct comparison with published studies on orbital and lacrimal gland diagnoses, we selected our study intervals close to those reported in the literature (Table 6). With the exception of studies from Naples and Tokyo, relative frequency of *dacryoadenitis* among lacrimal gland diagnoses in Freiburg was lower than in other centres: 11% (2005–2015) compared with 50% in Leeds (2003–2015) [50] and 18% in Dublin (2008–2018) [51], and 23% (1945–1995) compared to 57% in Atlanta (1941–1995) [10, 19]. Kennedy reported a frequency of 27% in Rochester (1949–1983) [18], closely aligning with our finding of 23% for 1945–1984. Conversely, Philadelphia reported a substantially higher frequency (54%, 1962–1982 vs. 28% in Freiburg, 1965–1984) [10, 19], while Naples (27%, 1976–2011 vs. 39% in Freiburg, 1975–2015) [49] and especially Tokyo (3.8%, 1983–2002 vs. 45% in Freiburg, 1985–2004) [53] documented lower proportions. The higher relative frequency of dacryoadenitis reported in [10, 18, 19, 50, 51] may reflect differences in biopsy indications for suspected cases. The 12-fold lower relative frequency (3.8%) reported in Tokyo [53] may reflect differences in the prevalence of underlying causes—such as viral infections (e.g., mumps, now less common due to widespread vaccination) and autoimmune diseases (e.g., Sjögren’s syndrome, IgG4-related disease)—as well as variations in biopsy rates, diagnostic criteria, or reporting practices between Japan and Western countries.

*Pleomorphic adenoma* is a slow-growing, painless tumour composed of a mixture of epithelial and stromal (connective tissue) elements, resulting in its “pleomorphic” (varied) microscopic appearance. It is the most common primary tumour of the lacrimal gland in adults. Although benign, incomplete excision may lead to recurrence, and—if left untreated—it may undergo malignant transformation into carcinoma ex pleomorphic adenoma. Complete surgical excision, including the lacrimal gland, is the standard treatment to prevent recurrence. Comparative studies reveal largely consistent findings between centres: Rochester (27%, 1949–1983 vs. 21% in Freiburg, 1945–1984) [18], Philadelphia (18%, 1962–1982 vs. 27% in Freiburg, 1965–1984) [10, 19], and Houston (7.4%, 1998–2009 vs. 7.3% in Freiburg, 1995–2004) [52] (Table 6). However, the notably lower proportion in Atlanta (3.1%, 1941–1995) [10, 19], compared with Freiburg (24%, 1945–1995), is initially surprising. This discrepancy may be explained by the fact that Emory University’s L.F. Montgomery Ophthalmic Pathology Laboratory is not an orbital referral centre, potentially influencing case selection and reported frequencies. In contrast, studies from Naples (32%, 1976–2011 vs. 15% in Freiburg, 1975–2015) [49], Leeds (25%, 2003–2015 vs. 4.4% in Freiburg, 2005–2015) [50], and Tokyo (64%, 1983–2002 vs. 18% in Freiburg, 1985–2004) [53] report significantly higher relative frequencies of pleomorphic adenoma diagnoses. The lower proportions observed in Freiburg reflect a consistently low number of cases across the last four study intervals, despite a moderate increase in the total number of lacrimal gland specimens: 4/24 cases (17%) in 1975–1984, 6/19 cases (32%) in 1985–1994, 4/37 cases (11%) in 1995–2004, and 2/45 cases (4.4%) in 2005–2015 (Table 1). The marked decline in the proportion of lacrimal gland pleomorphic adenomas observed in Freiburg since 1985—of uncertain aetiology—stands in contrast to the findings reported in studies from Naples, Leeds, and Tokyo. Existing literature indicates that the prevalence of lacrimal gland pleomorphic adenomas has either declined or remained stable over recent decades, whereas that of inflammatory and lymphoproliferative lesions has increased [59, 60].

### Lacrimal duct system

*Dacryocystitis* was the most frequent diagnosis, accounting for 72% of cases (23/32) in 1945–1954 (Table 1). Its proportion rose to 89% (42/47) in 1955–1964, then declined to 50% (11/22) in 1965–1974 and further to 40% (27/67) in our final study interval (2005–2015). These trends align with findings from Denmark: In a clinicopathological study of 643 lacrimal drainage system biopsy specimens (1910–1999) from the Eye Pathology Institute, University of Copenhagen, and the Danish Pathology Database (1970–1999), Marthin et al. [61] similarly identified dacryocystitis as the predominant diagnosis, representing 79% of cases. The significant drop in the relative frequency of dacryocystitis within the lacrimal duct system in our study—from 89% to 50% and below after 1965—aligns with the introduction of dacryocystorhinostomy and the routine use of antibiotics, which together enabled more effective and sustainable treatment of dacryocystitis. The decline in dacryocystitis diagnoses coincides with a rise in the proportion of *canaliculitis* cases, from 14% (3/22) in 1965–1974 to 30% (20/67) in 2005–2015, with fluctuations in the intervening periods (Table 1). This shift can be attributed to several key factors: an aging population (as canaliculitis is more common in middle-aged and older individuals), the increased use of medical and cosmetic interventions affecting the lacrimal system, evolving treatment protocols—including the adoption of minimally invasive techniques such as canaliculostomy, which facilitates the extraction and histological examination of canalicular dacryoliths—as well as a rise in risk factors such as punctal plugs for dry eye treatment [62] and persistent bacterial infections. Malignancies were rare across all intervals, with melanoma accounting for 9.1% of cases (2/22) in 1965–1974. Lymphoma appeared among the “Top 10” diagnoses only in the last two intervals (1995–2015), representing 5.7% and 4.5% of cases, respectively.

### Uvea and intraocular tissue, unspecified

*Iris melanoma* was the most frequent diagnosis throughout our study period, accounting for 17% of uveal pathologies. Early ophthalmology texts, such as those by Albrecht von Graefe (1828–1870) [63], describe iridectomy as a core surgical skill, well before the introduction of the operating microscope in the mid-twentieth century. It is therefore not surprising that iris specimens—with *iritis* being the predominant diagnosis in the earliest interval (39%, 1945–1954)—were excised in Freiburg already during the pre-surgical microscope era (Table 1).

Trauma-associated uveal tissue was categorized under “*intraocular tissue, unspecified*” (which also includes other intraocular components besides the iris). While only 2 cases were recorded in 1965–1974 and 1 case in 1975–1984, a marked increase was observed in the last three intervals: 43 cases (100%) in 1985–1994, 81 cases (99%) in 1995–2004, and 68 cases (99%) in 2005–2015 (Table 1). This trend likely reflects changes in the management of prolapsed tissue—specifically, an increased use of histological examination since the 1980s to identify retinal involvement (a negative prognostic factor for functional outcome and eye preservation [64])—rather than a true increase in perforating bulbar trauma. The observed increase in the median age of patients undergoing surgical removal of *intraocular tissue* due to trauma since 1965, with 17.5 years in 1965–1974 raising to 63.5 years (without retina) and 72 years (with retina) in 2005–2015 can be attributed to several key factors: We postulate that aging population, safer environments for younger people, improved imaging and diagnostics, and advances in medical and surgical care (vitrectomy, microsurgery, and intraocular repair techniques) have collectively shifted the age distribution of patients requiring intraocular trauma surgery toward older adults.

### Vitreous

Vitreous specimens present unique considerations in both surgery and histological examination due to the gel-like, avascular nature of the vitreous humour. In Freiburg, vitrectomy was rarely performed in the early decades, with only 1 case in 1955–1964 and 2 cases in 1965–1974. The introduction of pars plana vitrectomy (PPV) by Robert Machemer (1933–2009) in 1971–1972, however, represented a paradigm shift. Advances in this technique—alongside improved microsurgical instrumentation and anaesthesia—made vitrectomy both safer and more widely accessible. Consequently, the number of vitrectomies increased substantially across our last four study intervals, with *vitritis* emerging as the most frequent diagnosis: 3/6 cases (50%) in 1975–1984, 13/25 cases (52%) in 1985–1994, 36/73 cases (49%) in 1995–2004, and 32/71 cases (45%) in 2005–2015 (Table 1). The marked increase in the number of specimens between the fourth-last and third-last intervals likely reflects further refinements in vitrectomy techniques. In Freiburg, vitritis accounted for 47% of vitreous pathologies (84 of 178 cases) between 1945 and 2015. Analysis of combined vitreous and retinal specimens from 1945 to 1994 revealed a lower, though still substantial, proportion of 34%—significantly exceeding the 7.2% reported by Spraul and Grossniklaus for similar localisations in Atlanta (1941–1995) [10]. An analysis of our fourth and fifth time period (1975–1994) showed vitritis in 48% of vitreous cases (15/31), while Palexas et al. [65] documented a 40% frequency among 405 consecutive pars plana vitrectomies at The Johns Hopkins Medical Institutions, Baltimore, MD, USA (1973–1994). Between 1985 and 2004, the relative frequency of vitritis in Freiburg increased to 50% (49 of 98 cases), closely mirroring the 54% reported by Wittenberg et al. [66] in 278 diagnostic vitrectomies at Vancouver General Hospital, BC, Canada (1990–2005).

The increase in the median age of patients undergoing vitrectomy in Freiburg—evident in vitritis cases, where the age rose from 32.5 years in 1975–1984 to 73 years in 2005–2015 (Table 2)—reflects a broader shift in the procedure’s indications. Once primarily performed for trauma or congenital conditions, vitrectomy is now predominantly driven by age-related degenerative diseases, macular holes, epiretinal membranes and suspected lymphoma.

*Lymphoma* was the second most frequent diagnosis in our series, comprising 9.6% of vitreous cases (17/178) from 1945 to 2015. Strikingly, no cases were identified before 1995; however, its proportion increased to 5.5% (4/73) between 1995 and 2004, and further to 18.3% (13/71) in 2005–2015—though 7 of these later diagnoses were unconfirmed (Table 1). Consistent with our findings—where no cases were found (1945–1994)—lymphoma accounted for less than 0.5% of vitreous cases in Atlanta (1941–1995) [10]. In contrast, a 10% frequency was reported in Baltimore (1973–1994) [65]. Our own data for 1985–2004, however, revealed lymphoma in 4.1% of cases (4/98), closely aligning with the 5% relative frequency documented in Vancouver (1990–2005) [66].

Haemorrhage—resulting in the presence of *blood*—occurred during vitrectomy in 15 of 271 cases (5.5%) in our study. Specifically, 6.2% (11/178) of haemorrhages were vitreous-based, while 4.3% (4/93) involved the retina. The distribution across study intervals was as follows: 14% (1 of 7 cases) in 1975–1984, 15% (5 of 34 cases) in 1985–1994, 5.6% (6 of 107 cases) in 1995–2004, and 2.6% (3 of 117 cases) in 2005–2015 (Table 1). For comparison, a substantially higher relative frequency of 23.2% was reported in Atlanta (1941–1995) [10], likely due to inclusion of enucleated bulbi, whereas Freiburg recorded a proportion of 13% between 1945 and 1995. In contrast, Baltimore (1973–1994) documented only 1.0% [65]. Our findings, however, align closely with data from Vancouver, where haemorrhage was observed in 8.6% of cases (1990–2005) compared to our 7.1% (1985–2004) [66].

The apparent absence of *persistent hyperplastic primary vitreous* (PHPV) diagnoses in Freiburg before 1975 is unlikely due to a sudden emergence of the condition itself. Instead, it is rather a reflection of improved surgical technique. It accounted for 4.5% of vitreous cases (8 of 178 specimens), ranking as the fourth most frequent diagnosis in our series from 1945 to 2015. The proportion of cases decreased over time: 17% (1/6) in 1975–1984, 8.0% (2/25) in 1985–1994, and 6.8% (5/73) in 1995–2004 (Table 1). No cases were identified in our most recent study period. For comparison, Palexas et al. reported a 1.5% proportion of PHPV among vitreous diagnoses in Baltimore (1973–1994) [65], whereas in Freiburg, 9.4% of specimens (3/31) underwent histological analysis between 1975 and 1994.

### Retina

Retinal specimens are obtained during surgeries involving direct retinal manipulation, biopsy, or excision. This includes peeled epiretinal membranes (fibrocellular or fibrovascular) and subretinal tissue removed in cases of choroidal neovascularisation (CNV) or subretinal fibrosis. While retinal specimens were approximately half as frequent as vitreous samples in our series, their numbers increased during the 1985–1994 interval and beyond (Table 1), coinciding with advancements in pars plana vitrectomy techniques. In Freiburg, *fibrocellular or fibrovascular membranes* represented 45% of retinal pathologies (42 of 93 cases) analysed histologically between 1945 and 2015, though only 8.5% (4 of 47) were observed during the first four intervals (1945–1994). By contrast, Atlanta reported a significantly higher proportion of 72.9% for the comparable period (1941–1995) [10]. This nearly 9-fold higher number of fibrocellular or fibrovascular membrane specimens likely reflects differences in institutional practices including specialisation or research focus, clinical volume, patient demographics, and healthcare system infrastructure between the two centres. In Freiburg’s last three study intervals, proliferative vitreoretinopathy accounted for 33% (3 of 9 cases), 12% (4/34), and 13% (6/46) of retinal specimens, respectively (Table 1). Epiretinal gliosis contributed 13% (1 of 9 cases), 18% (6/34), and 20% (9/46) during the same periods. Additionally, unspecific membranes were identified in 8.8% of cases (3/34) between 1995 and 2004. In the most recent interval, proliferative diabetic membrane was observed in 13% of cases (6/46), and scar tissue in 8.7% (4/46).

*Choroidal neovascular membrane* (CNV) was the second most common diagnosis in our series, representing 23% of retinal cases (21/93) over the study period (Table 1). Its prevalence peaked at 41% (14 of 34 cases) in the penultimate interval but declined to 15% (7/46) in 2005–2015, coinciding with the introduction of antiangiogenic intravitreal injections in 2002 [67]. *Granuloma,* identified only in the final interval, accounted for 8.7% of cases (4 of 46) and represented the third most frequent diagnosis overall (4.3%, 4 of 93 cases). *Normal retinal tissue* was observed in 2.1% of cases (2 of 93), being the fourth most common diagnosis with one case each occurring in the 1965–1974 and 1985–1994 intervals. The proportion of normal retina specimens (2.3%, 1 of 43) during the third and second-last periods (1985–2004) closely corresponds to the 4.0% relative frequency reported by Wittenberg et al. in Vancouver (1990–2005) [66].

### Sclera, anterior chamber angle and anterior chamber

The *sclera*, the fibrous outer layer of the eyeball, provides essential structural protection and support to the eye. Scleral specimens are rarely submitted for histological analysis, as scleral pathologies—such as scleritis, infections, tumours, or degenerative conditions—are relatively uncommon. When suspected, these conditions are typically evaluated using slit-lamp examination and monitored with ultrasound biomicroscopy (UBM) to avoid the risks associated with biopsy. Over the observation period, the number of cases rose gradually from 1 in 1955–1964 to 13 in 2005–2015 (Table 1), a trend that likely reflects the steady growth in patient volume at the Freiburg Eye Center. Of 38 cases, 12 (32%) were normal sclera, 10 (26%) were scleritis, 5 (13%) were foreign body or foreign body granuloma, 3 (7.9%) were filtering bleb revision, and 2 (5.2%) were sclera plaque.

Trabeculectomy became the gold standard for glaucoma treatment in the 1970s and 1980s. This procedure involves excising a portion of the trabecular meshwork and adjacent angle structures to lower intraocular pressure. In Freiburg, Hanns-Hellmuth Unger’s research from the 1950s to the early 1980s focused on the chamber angle anatomy and trabecular meshwork—structures central to glaucoma pathophysiology. Initially, *anterior chamber angle* specimens were collected primarily for research purposes. The first 3 specimens (sclera and trabecular meshwork) were documented in histological routine diagnostics between 1965 and 1974, representing 8.1% of the 37 total cases submitted during the 1965–2015 period (Table 1). Their numbers peaked at 21 specimens (57%) in 1975–1984, then declined to 7 (19%) in 1985–1994, with none recorded in 1995–2004 and 6 specimens (16%) in the final interval. The most frequent findings were sclera and trabecular meshwork (19 cases, 51%), followed by inflammation (3 cases, 8.1%), and connective tissue, hemosiderin, iridodialysis, and pigmented trabecular meshwork (2 cases each, 5.4%). With 27 cases within our series, *anterior chamber* specimens were even more scarce and unevenly distributed across our observation period. Epithelial downgrowth was the most frequent diagnosis with 14 cases (52%), followed by 7 cases of inflammation (26%) and 3 cases of bleeding (11%) (Table 1). To our knowledge, no comparative data exist in the literature to contextualise our findings regarding the sclera, anterior chamber angle, and anterior chamber.

### Optic nerve

Before 1965, optic nerve biopsies were rarely performed due to procedural risks, limited therapeutic benefit, and inadequate diagnostic tools. However, advancements in diagnostic and surgical techniques, along with evolving clinical requirements, increased their feasibility and value after 1965. Of the 15 optic nerve biopsies in our series, only 1 case (6.7%) occurred in 1955–1964, while 5 cases (33%) were identified in both 1965–1974 and 1995–2004, and 4 cases (27%) in 1975–1984. The most common findings were *atrophy*, *glioma*, *meningioma*, and *normal tissue*, each observed in 3 cases (20% per diagnosis), followed by *malignant nerve sheath tumours* (2 cases, 13%) and *postoperative gliosis* (1 case, 6.7%). While optic nerve pathologies accounted for 1.8% of all surgically obtained specimens in Atlanta (1941–1995) [10], this high frequency likely reflects the inclusion of enucleated eyes. A meaningful comparison with the Freiburg archive is not possible, as optic nerve samples there exclude bulbi, comprising only 0.035% of all cases.

### Exenteration

Exenteration is a radical procedure involving the removal of orbital contents—including the eyeball, extraocular muscles, and fat—sometimes extending to adjacent structures such as eyelids or bone. It is reserved for life-threatening or locally aggressive diseases when other treatments are ineffective. At the beginning of our series (1945) until about 1960, total exenteration was standard, with high morbidity due to limited reconstructive options. From the early 1960s until the early 1990s, subtotal exenteration (preserving eyelids or bone) emerged for less aggressive tumours, with improved outcomes through skin grafts and flaps. Of the 59 exenterations identified in the Freiburg series, 4 to 9 cases (6.8% to 15%) occurred in the first four study intervals, while a peak of 15 cases (25%) was reached in the 1985–1994 period (Table 1).

Since the early 1990s, less invasive techniques, such as eyelid-sparing exenteration, are used for select cases. Advances in reconstruction—including microvascular free flaps and 3D-printed implants—have enhanced post-operative quality of life. Furthermore, the introduction of systemic therapies—such as BRAF inhibitors, immunotherapy, and the hedgehog pathway inhibitor vismodegib (approved in 2012) [68]—has significantly reduced the need for exenteration. Consequently, both the number and relative frequency of exenterations declined to 8 cases (14%) in each of the last two intervals (Table 1), despite a rise in total histological specimens submitted—from 862 in 1995 to 2,046 in 2015. In our series, the most common diagnoses indicating exenteration were *basal cell carcinoma* (BCC; 10 cases, 17%), *squamous cell carcinoma* (SCC; 6 cases, 10%), *lymphoma* (5 cases, 8.5%), *metastasis* (4 cases, 6.8%) and *sebaceous gland carcinoma* (SGC; 4 cases, 6.8%), followed by *melanoma*, *resection after R1* and *rhabdomyosarcoma,* each observed in 3 cases (5.1% per diagnosis).

We compared our findings with four retrospective studies from the literature, selecting Freiburg study intervals that most closely matched the published periods: Kuo et al. [69] analysed medical records of 38 patients who underwent orbital exenteration at Royal Prince Alfred and Concord Hospitals, Sydney, Australia (1990–2008). Soysal [70] reviewed 68 exenteration cases at Ankara Numune Education and Research Hospital, Ankara, Türkiye (1997–2007). Ali et al. [71] evaluated records of 119 patients who underwent orbital exenteration at L.V.Prasad Eye Institute, a tertiary eye care centre in Hyderabad, South India (1999–2012). Martel et al. [72] assessed records of 35 exenteration cases at Jules Gonin Eye Hospital, a specialised ocular oncology centre in Lausanne, Switzerland (2003–2017).

In Freiburg, the relative frequency of BCC as an indication for orbital exenteration aligns with international data: 30% (1985–2004) (Table 1) compared to 32% in Sydney (Kuo et al. [69]); 6.3% (1995–2015) versus 2.5% in Hyderabad (Ali et al. [71]); and 13% (2005–2015) compared to 8.0% in Lausanne (Martel et al. [72]. However, Soysal [70] reported a notably higher proportion of 38% in Ankara (1997–2007), likely due to late diagnoses and inadequate healthcare logistics, while no cases of BCC indicating exenteration were recorded in Freiburg (1995–2004).

The relative frequency of SCC requiring exenteration was markedly higher in Sydney (50%; 1990–2008 [69]) and Ankara (46%; 1997–2007 [70]), compared to Freiburg’s 8.7% (1985–2004) and 0% (1995–2004) (Table 1). These differences likely stem from delayed diagnoses and healthcare access issues, leading to more advanced disease and increased exenteration rates. Conversely, Freiburg’s data align closely with findings from Hyderabad (4.2%; 1999–2012 [71] vs. 6.3%, 1995–2015) and Lausanne (16%; 2003–2007 [72] vs. 13%, 2005–2015).

SGC accounted for a lower proportion of exenterations in Ankara (2.9%; Soysal et al. [70]) and Lausanne (12%; Martel et al. [72]) than in Freiburg (13%; 1995–2004 and 25%; 2005–2015, respectively). The results from Hyderabad (17%; Ali et al. [71]), however, closely correspond to our findings (19%; 1995–2015).

Our analysis revealed marked geographic variation in the relative frequency of melanoma as an indication for orbital exenteration. In Sydney—located 1,573 km closer to the equator than Freiburg—Kuo et al. [69] reported a melanoma rate of 13%, compared to only 8.7% in Freiburg (1985–2004). By contrast, Soysal [70] documented a lower rate of 4.4% in Ankara—located 898 km closer to the equator—compared to Freiburg’s 25% (1995–2004). The disparity was even more pronounced in Hyderabad—located 3,408 km closer to the equator—where Ali et al. [71] observed a melanoma rate of just 0.84%, likely due to the higher levels of cutaneous pigmentation in the population, while ocular surface squamous neoplasia (OSSN) dominated at 40%, against Freiburg’s 19% (1995–2015). The high frequency of OSSN in Hyderabad (40%) likely reflects the combined effects of intense and prolonged UV radiation exposure in the region, given its geographic proximity to the equator. While cutaneous pigmentation may offer some protection against skin cancers, it does not shield the conjunctiva and cornea—tissues directly exposed to solar UV rays. Additionally, chronic irritative factors such as dry eye disease, dust, and environmental pollutants may further contribute to the development of OSSN by promoting persistent inflammation and cellular damage. In contrast of the findings in Hyderabad, melanoma was the predominant indication in Lausanne (56%; Martel et al. [72]), likely reflecting the centre’s specialization in ocular oncology and the use of advanced therapies for BCC, whereas Freiburg recorded 13% (2005–2015). These disparities may be attributed to geographical and demographic differences, ethnicity and, where applicable, degree of skin pigmentation, the specialisation of treatment centres, and advancements in diagnostic and therapeutic practices over time.

### Limitations and strengths

This study presents a large retrospective analysis of 43,169 histological diagnoses derived from 39,256 archived specimens spanning 1945 to 2015. A substantial proportion of specimens underwent microscopic re-evaluation and analysis by experienced ophthalmic pathologists. Nonetheless, this study is subject to several limitations. Our analysis was restricted to specimens obtained through ophthalmic surgical intervention, which inherently introduces selection bias. Key sources of potential bias and variation include shifts in therapeutic standards, surgical techniques, and institutional capacities, as well as the ophthalmologist’s decision to operate, patient willingness to undergo surgery, and the selection of specimens for histopathological assessment. Changes in the hospital’s geographic catchment area and demographic trends may further affect the generalisability of our findings. These factors collectively constrain the epidemiological interpretation of disease frequency, prevalence, and severity in the broader population. Additionally, as a large tertiary referral centre, our institution may overrepresent patients with rare ocular conditions, complex or multiple diagnoses, and diseases resistant to conventional therapies. The extensive service area of the Freiburg Eye Center may also lead to disparities in patient representation, particularly among elderly individuals living at greater distances compared to those residing nearby.

A defining strength of this study is its uninterrupted 71-year dataset, allowing correlations between historical events (e.g., post-war reconstruction, the 1964 opening of the Freiburg Eye Center), technological advancements (e.g., microsurgery, DMEK, vitrectomy), and diagnostic trends. By analysing data across distinct time periods rather than as an overall incidence, we enhance comparability with other studies and enable clearer associations between observed trends and historical developments. The dataset includes comprehensive information on patient age, surgery dates, and histopathological diagnoses. Additionally, the well-documented history of ophthalmology and ophthalmic pathology in Freiburg allows us to contextualise trends in diagnostic frequency with historical events, advancements in surgical techniques, and emerging treatment options. We further validated our findings by comparing them with 38 international studies involving samples with histological diagnoses from various ocular topographies, discussing both similarities and differences. Together, these data contribute to a broader understanding of the types and relative frequencies of ocular conditions requiring surgical excision and histopathological examination.

## Conclusions

This study reviewed 43,169 histopathological diagnoses from 39,256 specimens collected over 71 years (1945–2015) at the Eye Center, University of Freiburg, to explore long-term trends in ophthalmic pathology. The eyelid (50%) was the most frequent site, with chalazion, BCC, and papilloma as leading diagnoses, followed by Fuchs’ dystrophy, keratoconus, and keratitis in the cornea (17%), while pterygium and nevus prevailed in the conjunctiva (14%). In the orbit (1.2%), inflammation and lymphoma were most common. Key trends included a rise in chalazion, Fuchs’ dystrophy, and pterygium, driven by surgical innovations (e.g., microsurgery, DMEK) and increased UV exposure, while SCC declined and BCC diagnoses occurred at younger ages, likely due to improved UV protection and earlier detection. The expanding age range of patients across most diagnoses reflects aging populations and broader surgical indications.

Comparisons with 38 international studies confirmed regional variations, such as higher rates of chalazion and BCC in Freiburg than in Asian cohorts, likely due to differences in UV exposure, genetic predisposition, and healthcare practices. The rise in orbital lymphoma and vitritis aligns with global trends, underscoring the role of advanced imaging and histopathological refinement in diagnosis. The study highlights the critical role of ophthalmologists in pathology, ensuring seamless integration of clinical and histopathological expertise for accurate diagnoses and optimal patient outcomes.

Outlook: As ophthalmic pathology becomes increasingly specialised, its integration with clinical ophthalmology remains vital for precision medicine, research, and training future specialists. Future research should focus on molecular diagnostics, personalised therapies, and global comparisons to drive progress. Our findings underscore the importance of ophthalmologist-led pathology in linking clinical and histological diagnosis, thereby enhancing both high-quality, patient-centred care and scientific innovation in the field.

## Data Availability

The datasets used and/or analysed during the current study are available from the corresponding author on reasonable request.

## Abbreviations

BCC: Basal cell carcinoma
BK: Bullous keratopathy
CIN: Conjunctival intraepithelial neoplasia
CNV: Choroidal neovascularisation
DMEK: Descemet membrane endothelial keratoplasty
DOP: Association of German-speaking Ophthalmic Pathologists
EMZL: Extranodal marginal zone B-cell lymphoma
EOPS: European Ophthalmic Pathology Society
HPV: Human Papillomavirus
IHC: Immunohistochemistry
NHL: Non-Hodgkin lymphoma
OSSN: Ocular surface squamous neoplasia
PHPV: Persistent hyperplastic primary vitreous
PKP: Perforating keratoplasty
PPV: Pars plana vitrectomy
PVR: Proliferative vitreoretinopathy
SCC: Squamous cell carcinoma
SGC: Sebaceous gland carcinoma
UBM: Ultrasound biomicroscopy
VZS: Verhoeff-Zimmermann Society
